# Experimental and theoretical study for CO_2_ activation and chemical fixation with epoxides†

**DOI:** 10.1039/c8ra10475a

**Published:** 2019-04-29

**Authors:** Jinwei Gao, Liuyi Li, Caiyan Cui, Muhammad Asad Ziaee, Yaqiong Gong, Rongjian Sa, Hong Zhong

**Affiliations:** College of Science, North University of China Taiyuan Shanxi 030051 P. R. China; State Key Laboratory of Structural Chemistry, Fujian Institute of Research on the Structure of Matter, Chinese Academy of Sciences Fuzhou Fujian 350002 China zhonghong@fjirsm.ac.cn; Institute of Oceanography, Ocean College, Fujian Provincial Key Laboratory of Information Processing and Intelligent Control, Minjiang University Fuzhou Fujian 350108 China rjsa@mju.edu.cn

## Abstract

The synthesis of five-membered cyclic carbonates *via* catalytic cycloaddition reaction of CO_2_ with epoxides is considered to be an effective technology for alleviation of the energy crisis and global warming. Various commercial organic bases and ionic salts were used as catalysts, while the relationship of catalytic activity and compound structure has been seldom explored. Herein, a facilely obtained binary catalytic system based on triethylamine/NBu_4_Br was developed for CO_2_ activation and chemical fixation. The highly efficient catalytic system showed outstanding conversion and above 99% selectivity under metal-free mild reaction conditions (100 °C, 1 atm) in one hour. The detailed process of CO_2_ activation and chemical fixation was investigated at the molecular level by a series of experiments and theoretical calculation, which provided a mode for the design and synthesis of a highly efficient catalytic system for conversion of CO_2_ under mild conditions.

## Abstract 2

NEt_3_/NBu_4_Br works as an excellent metal-free catalyst for CO_2_ cycloaddition with epoxides and the detailed process of CO_2_ activation by NEt_3_ is first studied by theoretical calculation.

## Graphical abstract

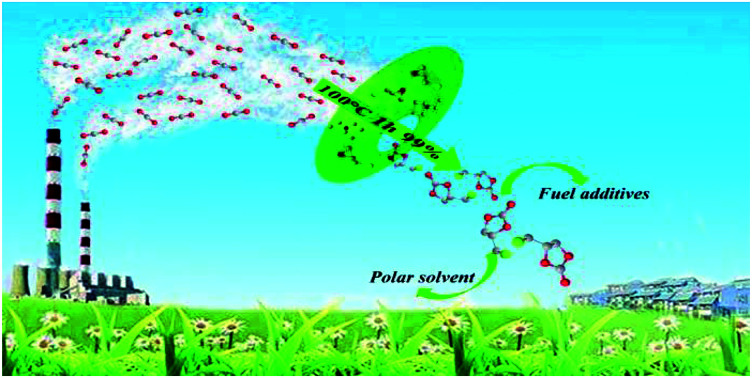

## Introduction

Carbon dioxide (CO_2_) excessive emission is a main component of greenhouse gases in the atmosphere.^1–3^ Its conversion to value-added products has offered one promising way to alleviate the energy crisis and global warming.^4–7^ As an economical and abundant C_1_ source, CO_2_ is renewable, inexpensive and non-toxic,^8–11^ but its chemical inertness is still the bottleneck for its applicability as a raw material in industry. The catalytic synthesis of cyclic carbonate from epoxides and CO_2_ is considered to be one of the most promising pathways to CO_2_ utilization.^12–14^ Cyclic carbonates have wide applications as green polar aprotic solvents, fuel additives, and chemical intermediates.^15–18^ The chemical fixations of CO_2_ into cyclic carbonates are relatively simple from a chemical reactivity point of view, while in fact, the high temperature, high pressure, high catalyst loading, or a combination of these are required to make the reaction effective, which is not economically suitable and poses safety concerns as well.^19–22^ To optimize the reaction conditions, a great variety of catalysts for the synthesis of cyclic carbonates have been developed so far, including alkali-metal halides, metal complexes, metal oxides, cellulose, MOFs, zeolites, ionic liquids, carbon nitride and so on.^23–32^ Though most catalysts were used to activate epoxide by a metal ion or hydrogen bond center, the effect of CO_2_ activity on the reaction efficiency has rarely been studied deeply.

CO_2_ is the highest oxidation state of carbon, and it is thermodynamically stable and kinetically inert, which will consequently hinder the development of efficient catalysts that achieve CO_2_ activation and subsequently its functionalization.^23,33,34^ Thus the activation of CO_2_ is pivotal for its effective conversion. The introduce/use of Lewis basic species and transition metal system have been highly considered, while the detailed CO_2_ activation process was unclear,^35–38^ so developing a catalytic system that provides molecular level insight for CO_2_ activation process is still highly desired. Taking the aforementioned concerns into account, we propose to explore a new catalytic system that can activate and convert CO_2_ with epoxides under mild conditions, and serve as an ideal mold for providing a detailed mechanistic understanding of CO_2_ activation and fixation process.

Herein, a simple and efficient binary catalytic system based on organobase/NBu_4_Br was developed for CO_2_ cycloaddition reaction with epoxides under metal-free mild conditions. It was found that the synergistic effect between two components in this new catalytic system promote the cycloaddition reaction occur under atmospheric pressure during a short time period of 1 hour. Moreover, the relationship of catalytic activity and catalyst structure was investigated at molecular level by a series of experiments and theoretical calculation, which could not only offer in-depth understanding of the reaction mechanism but also provide a theoretical basis for the effect of triethylamine (NEt_3_) in activating CO_2_ and promoting the reaction process.

## Results and discussion

To understand the effects of the counter anions on the catalytic activity, the catalytic cycloaddition reaction of CO_2_ with epoxides were initially investigated in the presence of 0.5 mL NEt_3_. As shown in Table 1, NBu_4_Br afforded a full conversion of 2-(chloromethyl)oxirane to 4-(chloromethyl)-1,3-dioxolan-2-one under 1.0 atm. CO_2_ at 100 °C for 1.0 h (entry 1), and the catalytic conversion showed unconspicuous change when the NBu_4_Br was replaced by NBu_4_Cl, NBu_4_I, respectively (entries 2–3). Notably, the yield of 2-(chloromethyl)oxirane to 4-(chloromethyl)-1,3-dioxolan-2-one decreased significantly when NBu_4_PF_6_ was used under the same condition, which is probably due to the weakest nucleophilicity of PF_6_^−^ in selected Cl^−^, Br^−^, I^−^, and PF_6_^−^, suggesting the counter anions play a dominant role in cycloaddition reaction of CO_2_ with epoxides.^39^ When the common organic base NEt_3_ was used alone under the same condition, 36% yield of 2-(chloromethyl)oxirane to 4-(chloromethyl)-1,3-dioxolan-2-one was obtained (entry 6), indicating that NEt_3_ can activate CO_2_ in this reaction.^40^ Based on the above results, the high efficiency of the binary system is probably attributed to a synergistic effect between NBu_4_Br and NEt_3_ during the catalytic conversion of CO_2_ with epoxides to cyclic carbonates.

**Table 1:** Catalyst screening for the conversion of CO_2_ with 2-(chloromethyl)oxirane^a^

Entry	Catalyst	Time (h)	Yield^b^ (%)	Select.^b^ (%)
1	NBu_4_Br + NEt_3_	1	99	>99
2	NBu_4_Cl + NEt_3_	1	97	>99
3	NBu_4_I + NEt_3_	1	96	>99
4	NBu_4_PF_6_ + NEt_3_	1	43	>99
5	NBu_4_Br	1	34	>99
6	NEt_3_	1	36	>99

a Reaction conditions: 2-(chloromethyl)oxirane (12.8 mmol), NBu_4_X (X = Br^−^, Cl^−^, I^−^, PF_6_^−^) (0.06 mmol), NEt_3_ (0.5 mL), CO_2_ (1 atm), 100 °C.

b Yield was determined by GC and ^1^H NMR. The possibility of by-product was 3-chloro-1,2-propanediol.

**Fig. 1:**
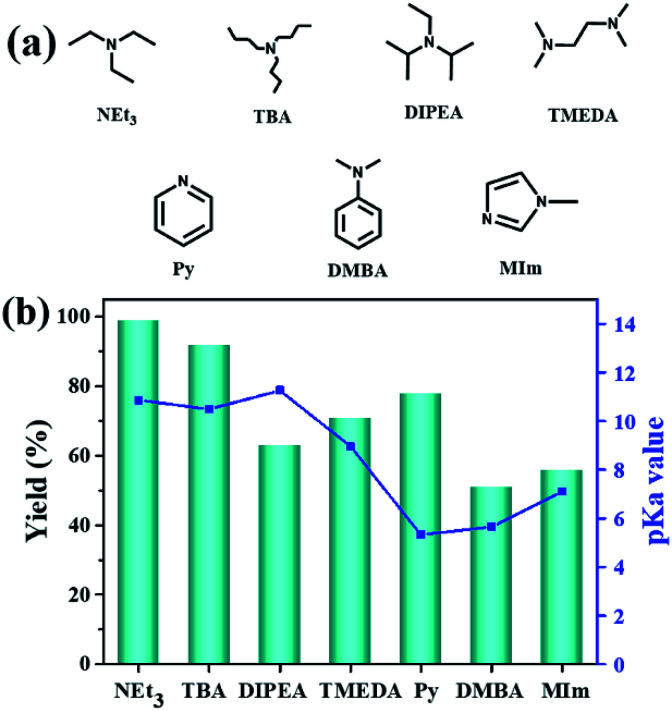
Organic bases structures (a); the effect of organic bases on the yield of cyclic carbonate (b). pKa value of the conjugated acid in water which was obtained from the U.S. National Library of Medicine database.^42^ Reaction conditions: 2-(chloromethyl)oxirane (12.8 mmol), NBu_4_Br (0.06 mmol), organic base (0.5 mL), CO_2_ (1 atm), 100 °C, 1 h. The possibility of by-product was 3-chloro-1,2-propanediol.

Additionally, the influence of the basicity on the conversion of 2-(chloromethyl)oxirane was investigated in the presence of NBu_4_Br with various organic bases at 100 °C for 1 h (Fig. 1a and b). As shown in Fig. 1b, the yield of 4-(chloromethyl)-1,3-dioxolan-2-one greatly related to the basicity of organic bases, and NEt_3_ gave rise to the highest conversion of 2-(chloromethyl)oxirane to corresponding product due to its strongest alkalinity and minimum steric hindrance. The activities of organic bases decreased sharply from 99% to 92 and 63% when the pKa decrease from 18.8 to 10.2, respectively. As reported previously, the base was weaker, the Δ*G* of this reaction was lower,^41^ so the basicity order might be DIPEA > NEt_3_ > TBA > TMEDA > MIm > Py > DMBA. However, the catalytic activity order of the organic bases is not in strict accordance with the established pKa, that was NEt_3_ > TBA > Py > TMEDA > MIm > DIPEA > DMBA, which indicates that the basicity of the organic bases is one important factor for promoting catalytic activity, but the steric-hindrance also play a role in this catalytic reaction.^23^

**Fig. 2:**
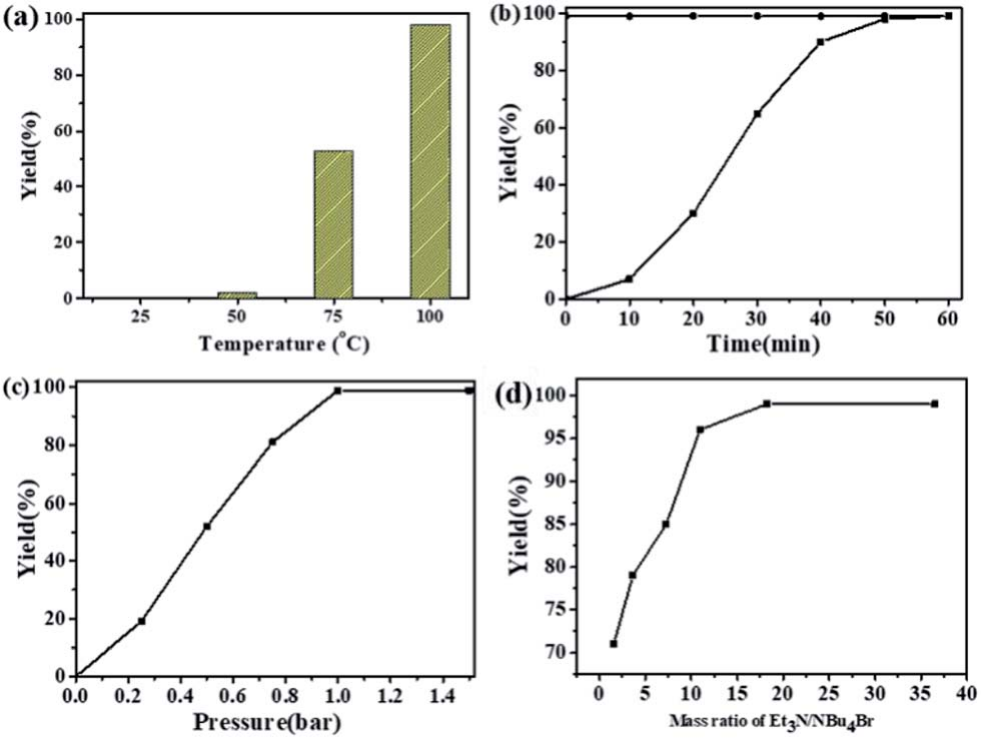
Effects of different reaction parameters. (a) Effects of reaction temperature, conditions: 2-(chloromethyl)oxirane (12.8 mmol), NBu_4_Br (0.06 mmol), NEt_3_ (0.5 mL), CO_2_ (1 atm), 1 h. (b) Effects of reaction time, conditions: 2-(chloromethyl)oxirane (12.8 mmol), NBu_4_Br (0.06 mmol), NEt_3_ (0.5 mL), CO_2_ (1 atm), 100 °C. (c) Effects of CO_2_ pressure, conditions: 2-(chloromethyl)oxirane (12.8 mmol), NBu_4_Br (0.06 mmol), NEt_3_ (0.5 mL), 100 °C, 1 h. (d) Effects of mass ratio of NEt_3_/NBu_4_Br, conditions: 2-(chloromethyl)oxirane (12.8 mmol), NBu_4_Br (0.06 mmol), CO_2_ (1 atm), 100 °C, 1 h. The possibility of by-product was 3-chloro-1,2-propanediol.

The dependence of the cycloaddition reaction of CO_2_ and 2-(chloromethyl)oxirane on temperature is shown in Fig. 2a. The results indicated that the activity of this catalytic system is highly dependent on the reaction temperature. In the lower temperature region (25 to 50 °C), the yield of 4-(chloromethyl)-1,3-dioxolan-2-one increases slowly with increasing temperature. A further increase in temperature from 50 to 100 °C has significant effects on the 2-(chloromethyl)oxirane conversion, and gave the target product in 99% GC yield at 100 °C for 1 h.

The kinetic curve for catalytic conversion of CO_2_ into cyclic carbonates was also investigated in the presence of NEt_3_/NBu_4_Br at 100 °C. As shown in Fig. 2b, the yield of 4-(chloromethyl)-1,3-dioxolan-2-one increased rapidly in the first 40 min and then went up slowly. The complete consumption of 2-(chloromethyl)oxirane and synchronous formation of the desired product was achieved in 1 h. It should mentioned that the selectivity remains above 99% in the entire catalytic process. It could be obviously seen that the reaction pressure showed a great effect on the cycloaddition reaction (Fig. 2c). With the increase of CO_2_ from 0.25 to 1 atm, the 4-(chloromethyl)-1,3-dioxolan-2-one yield increases from 20 to 99%. A further increase in the CO_2_ pressure from 1 to 1.5 atm results in a same level in 2-(chloromethyl)oxirane conversion. A similar effect of CO_2_ pressure on catalytic activity was observed in other related catalytic systems.^43,44^

The influence of NEt_3_ to NBu_4_Br ratio on the yield of 4-(chloromethyl)-1,3-dioxolan-2-one was also investigated at 100 °C for 1 h with fixed NBu_4_Br (0.06 mmol). As shown in Fig. 2d, when the ratio of NEt_3_ to NBu_4_Br increases from 1.6 to 18.2, the 4-(chloromethyl)-1,3-dioxolan-2-one yield increased rapidly. Then the 2-(chloromethyl)oxirane conversion stayed almost constant when ratio of NEt_3_ to NBu_4_Br increased further.

The excellent catalytic activity encouraged us to further explore the generality of the catalytic system, and several expoxides with different steric and electronic characters were tested. The results show that the various diverse epoxides were converted to the corresponding cyclic carbonates in high yields and excellent selective by this effective catalytic system (Table 2). The complete conversion of 2-(chloromethyl)oxirane is attributed to the presence of electron-withdrawing –CH_2_Cl group (entry 1), which facilitates nucleophilic attack of halide anions during the ring opening of the epoxide.^45,46^ The catalytic system is still effective for the epoxides with long alkyl carbon chain, such as 2-butyloxirane, 2-(ethoxymethyl)oxirane and 2-((allyloxy)methyl)oxirane (entries 2–4). The reactivity of epoxides with aromatic substituent is similar to that of long alkyl carbon chain (entries 5, 6). Interestingly, the complete conversion of 2,2′-(((propane-2,2-diylbis(4,1-phenylene))bis(oxy))bis(methylene))bis(oxirane) into 4,4′-(((propane-2,2-diylbis(4,1-phenylene))bis(oxy))bis(methylene))bis(1,3-dioxolan-2-one) could be achieved in 18 h (entry 7).

**Table 2:** Cycloaddition of CO_2_ to various expoxides catalyzed by triethylamine/NBu_4_Br^a^

Entry	Epoxide	Product	Time (h)	Yield^b^ (%)	Sel. (%)
1	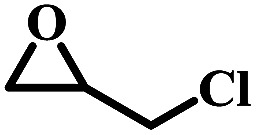	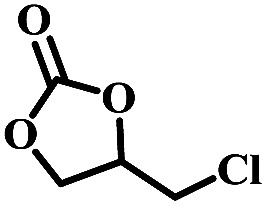	1	98	>99
2	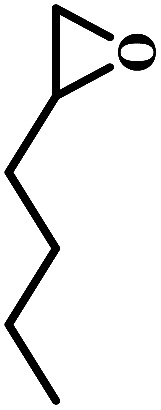	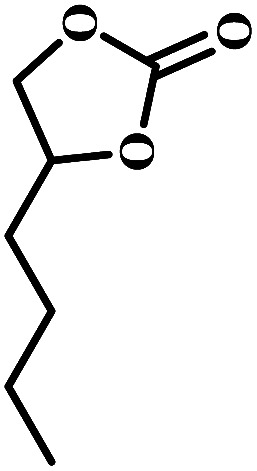	1	7	>99
			10	59	
			20	98	
3	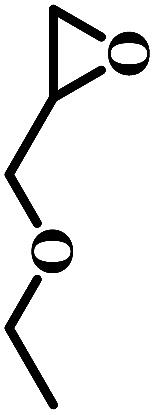	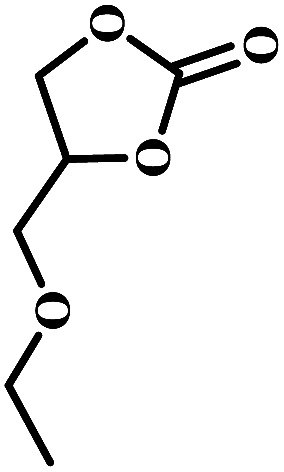	1	17	>99
			8	82	
			12	98	
4	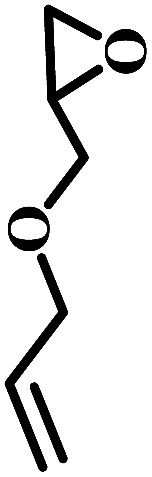	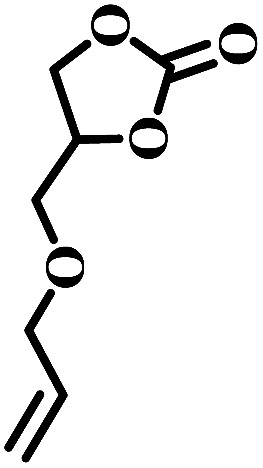	1	16	>99
			4	85	
			8	99	
5	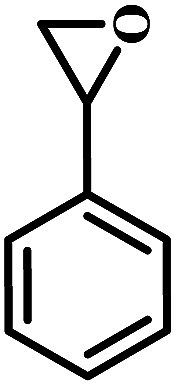	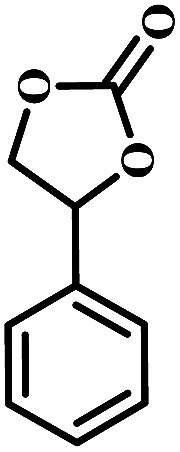	1	11	>99
			10	66	
			20	99	
6	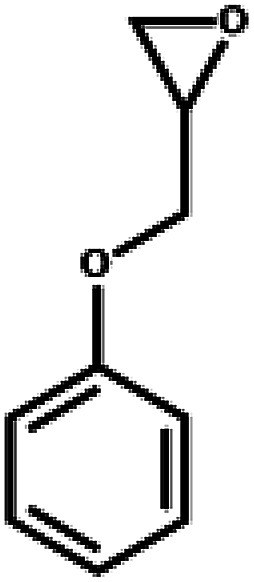	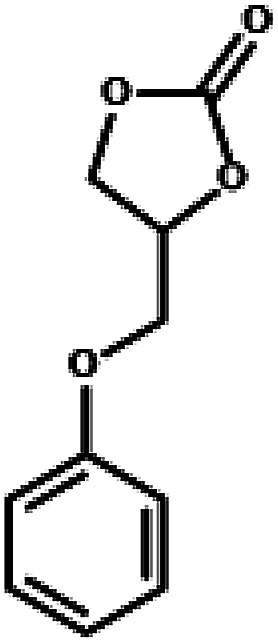	1	17	>99
			4	95	
			6	99	
7	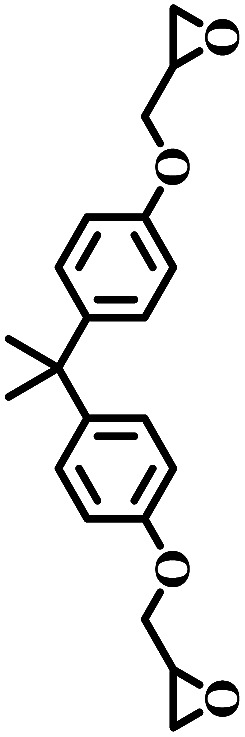	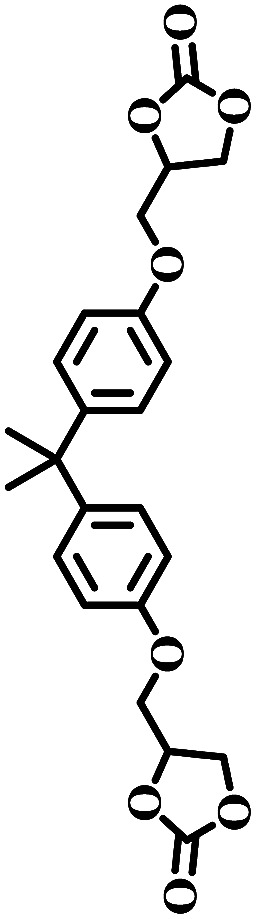	1	21	>99
			8	79	
			18	99	

a Reaction conditions: epoxide (12.8 mmol), NBu_4_Br (0.06 mmol), NEt_3_ (0.5 mL), CO_2_ (1 atm), 100 °C.

b Yield was determined by GC and ^1^H NMR. The possibility of by-products were corresponding diols.

**Fig. 3:**
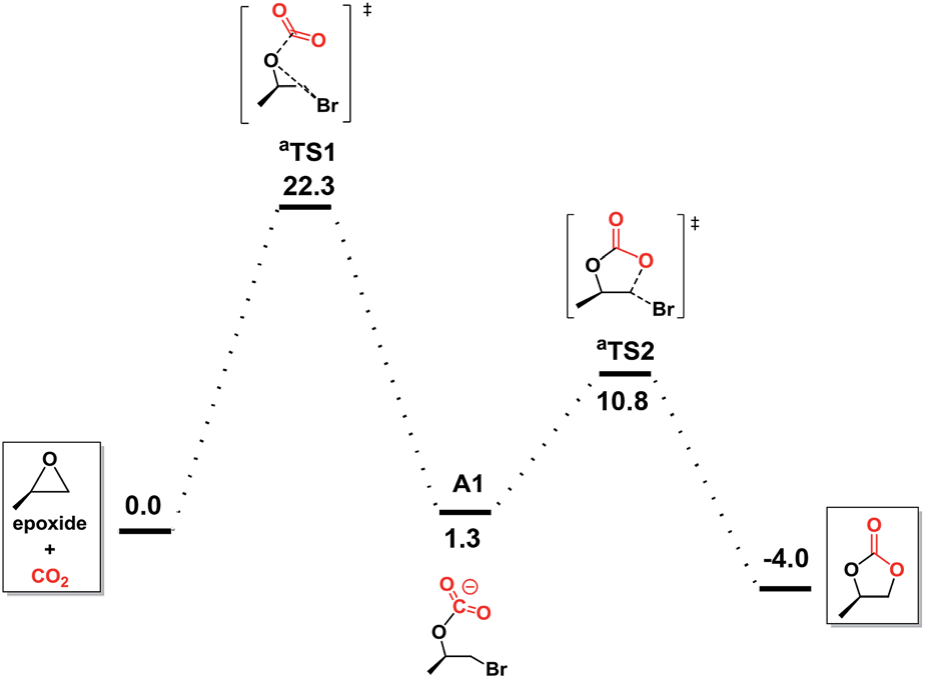
Free energy profiles for carbonate formation with bromine as catalyst in gas-phase.

To further understand the underlying principles of CO_2_ activation, the computation by the DFT (M06-2X) calculations was studied. Preliminary calculations indicated no involvement of NBu_4_^+^ cation (Fig. S1 in ESI†), which was consequently neglected from the elaborate calculations reported herein.^47^ With bromide as catalyst (Fig. 3), the ring opening through the attack of a nucleophile on epoxide and CO_2_ addition takes place simultaneously in ^a^TS1, which is considered to be the rate-determining step with a free energy of 22.3 kcal mol^−1^. Subsequently, this reaction tended to undergo the carbonate ring-closure step (^a^TS2) has the energy of 10.8 kcal mol^−1^ compared to the reactants. This is consistent with the inefficient reaction under the mild experimental conditions.

**Fig. 4:**
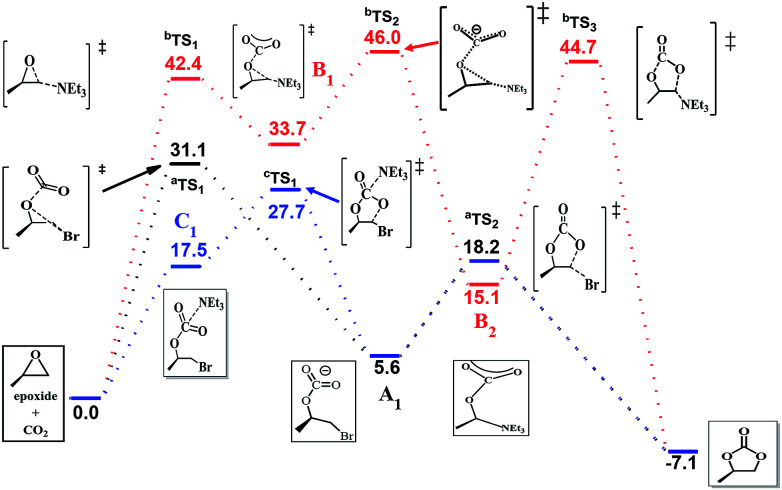
Free energy profiles of three different mechanisms in NEt_3_.

The efficiency of the reaction is promoted significantly when NEt_3_ is introduced as the catalyst in addition to the system. To gain further insight into the synergetic catalytic role played by NEt_3_ and NBu_4_Br, more detailed calculations were carried out. When the reaction was calculated in NEt_3_ solution, there different mechanisms were revealed (Fig. 4). The bromide-catalyzed process (black energy profile shown in Fig. 4) in NEt_3_ solution shows the same transition states and intermediates as in gas-phase. But the energy barrier of rate-determining step is 31.1 kcal mol^−1^, which is 8.8 kcal mol^−1^ larger this procedure without NEt_3_ solvent. Also the carbonate ring-closure step (^a^TS2) in NEt_3_ solution has an energy of 18.2 kcal mol^−1^, which is 7.4 kcal mol^−1^ larger than bromide-catalyzed process in gas. For comparison, we also calculated the reaction with NEt_3_ as catalyst in the absence of bromide (red profile). The NEt_3_-catalyzed epoxide ring-opening procedure has a high energy barrier (^b^TS1, 42.4 kcal mol^−1^, red profile in Fig. 4), which is 11.3 kcal mol^−1^ larger than bromide-catalyzed transition state in NEt_3_ solution. Afterwards, the CO_2_ addition step to form ^b^TS2 has an energy of 46.0 kcal mol^−1^ relative to the energy of the reactants. Finally, this mechanism of ring-closure process may undergo a ^b^TS3 transition states, which has an energy of 44.7 kcal mol^−1^ above the reactants. However, the energy barrier of 42.4 kcal mol^−1^ is quite high and consistent with a sluggish reaction and unable to take place under mild experimental conditions,^48^ indicating that NEt_3_ does not merely act as a solvent but also influences the course of the reaction. Thus, the mechanism when NEt_3_ participation tends to a bromine and NEt_3_ jointly catalyzed process (blue profile shown in Fig. 4), a new intermediate C_1_ appears, resulting from the CO_2_ addition upon interaction with NEt_3_ and bromine. This rate-determining step presented no transition states and has an energy of 17.5 kcal mol^−1^ the above reactants, 4.8 kcal mol^−1^ energy favorable than pure bromine-catalyzed process in gas-phase, 13.8 kcal mol^−1^ decrease than bromine-catalyzed procedure in NEt_3_ solvent, which allows the reaction to be performed under mild conditions. A potential energy surfaces scan calculation based on C_1_ intermediate validates transition states non-existent (Fig. S3 in ESI†). It is conceivable that the ring-opening of epoxide and CO_2_ addition might undergo synchronously with two catalysts. Afterwards, ring-closure takes place through ^c^TS1. NEt_3_ then dissociates from the reacting system, which reverts to A_1_ and evolves to product through ^a^TS2 with a free energy of 27.7 kcal mol^−1^ above the reactants. The calculated rate constant is 1.850 × 10^−3^ s^−1^ at 373 K. Then the corresponding half-life is ten minute, which accord with the experimental data. The intermediate of A_1_ with C_1_ were selected to compare the structure changes in reaction, the C–O bond of CO_2_ change slightly as shown in Fig. S4,† while the C–O bond between C atom of CO_2_ moiety and O atom of epoxide moiety decreases from 1.46 Å to 1.43 Å, then the interaction between CO_2_ and epoxide moieties enhanced with NEt_3_ as solution and catalyst. Moreover, the Mulliken charge of carbon atom of CO_2_ moiety decreases from 0.3 to 0.07 when compared C_1_ with A_1._ Therefore, the bond length and charge analysis suggest that the lone pair electron of nitrogen in NEt_3_ stabilized the formation of C_1_ and ^c^TS1.

**Fig. 5:**
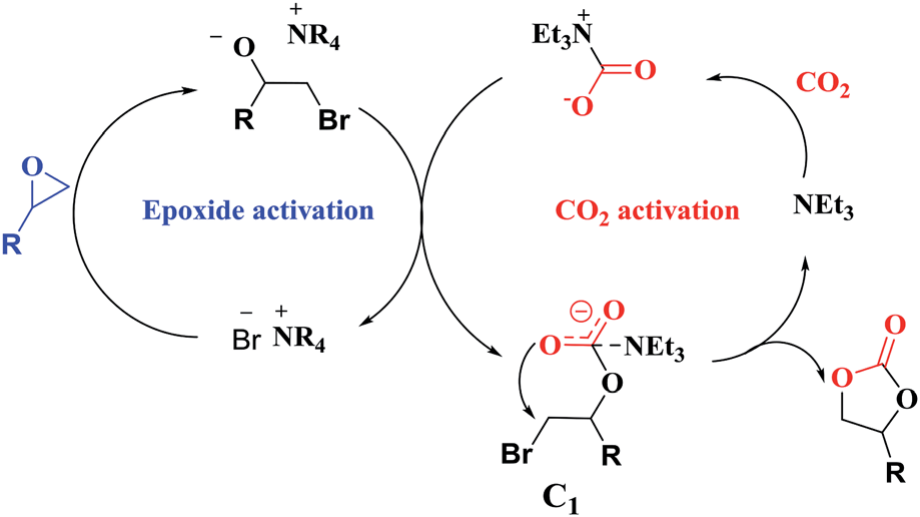
Proposed reaction pathway for cyclic carbonate formation catalyzed by NEt_3_ and NBu_4_Br.

Overall, a plausible mechanism for triethylamine-promoted catalytic conversion of CO_2_ into cyclic carbonates has been proposed based on the aforementioned results (Fig. 5). First, the epoxide ring opens through nucleophilic attack on the less sterically hindered β-carbon atom by Br^−^ to produce an alkoxide ion, and simultaneously CO_2_ is activated by NEt_3_*via* electrostatic interaction to form the carbamate salt. Then a new intermediate C_1_ is produced resulting from the nucleophilic attack on carbamate salt by alkoxide ion. Finally, cyclic carbonate is produced by following intramolecular ring-closure reaction of C_1_.

## Conclusions

An efficient binary catalytic system containing triethylamine and NBu_4_Br was screened for catalytic conversion of CO_2_ and epoxides into cyclic carbonates under metal-free mild conditions. Especially, NEt_3_ could activate CO_2_*via* electrostatic interaction and remarkably reduce the reaction energy to promote the reaction in the catalytic system. This work not only presents a simple and useful route for CO_2_ chemical fixation into high-value chemicals, but provides a detailed mechanistic understanding of CO_2_ activation and fixation process.

## Experimental

### General

Chemicals including NEt_3_, NBu_4_Br, NBu_4_Cl, NBu_4_I, NBu_4_PF_6_, epoxides and CO_2_ are commercially available and used directly without further purification. Gas chromatography (GC) was performed on a Shimadzu GC-2014 equipped with a capillary column (RTX-5, 30 m × 0.25 μm) using a flame ionization detector.

### Typical catalytic reaction

The cycloaddition reaction was carried out by magnetic stirring, trimethylamine (0.5 ml), NBu_4_Br (0.06 mmol) and 2-(chloromethyl)oxirane (12.8 mmol) were added into a reactor at room temperature. Then, the reactor was sealed and purged with CO_2_ to remove air. CO_2_ was introduced into the reactor and the pressure was adjusted to 1 atm at room temperature. The reactor was placed into pre-heated oil bath and temperature was maintained at 100 °C. After the reaction was completed, the reactor was cooled to 0 °C in ice-water bath, and then the excess of CO_2_ was carefully vented. The mixture was diluted with ethyl acetate. The conversion of epoxide and yield of cyclic carbonate were determined by gas chromatography (Shimadzu GC-2014, a flame ionization detector) and ^1^H NMR.

### Computational details

The M06-2X functional^49^ was employed in this article to perform all the calculations. Our structure optimizations were as follows. In NBu_4_Br involved reaction, only gas-phase calculations were performed. For carbonate formation with bromine as catalyst, the structures were firstly optimized base on the level of 6-31+G(d,p) in gas-phase, then the solvent structure optimizations were carried out based on gas-phase results. As for the rest of other structure optimizations, the calculations were performed in solvent. Vibrational frequency analyses at the same basis sets were used on all optimized structures in order to characterize stationary points as local minima or transition states. Furthermore, the intrinsic reaction coordinate (IRC) calculations at the same level have been applied to validate that transition states connect appropriate reactants and products. The Gibbs free energy were further calculated by single-point energy calculations using M02-2X/6-311+G(d,p) method on previously 6-31+G(d,p) structures and thermal corrections at 298.15 K and 1 atm. No conformational sampling calculations were performed in this work. The continuum SMD model^50^ was applied. The Gaussian 09 package^51^ was used for all of our calculations in NEt_3_ solvent.

## Conflicts of interest

There are no conflicts to declare.

## Supplementary Material

RA-009-C8RA10475A-s001
