# Supplementary material for: Experimental and theoretical study for CO2 activation and chemical fixation with epoxides
Source: RSC Adv. 2019 Apr 29;9(23):13122–7. doi: 10.1039/c8ra10475a (PMC9063868; doi:10.1039/c8ra10475a)
Supplement: RA-009-C8RA10475A-s001 [file RA-009-C8RA10475A-s001.pdf]

Electronic Supplementary Material (ESI) for RSC Advances.
This journal is © The Royal Society of Chemistry 2019

Electronic supplementary information

for

Experimental and theoretical study for CO_2 activation and chemical fixation with epoxides

Jinwei Gao,[a,b] Liuyi Li,[b] Caiyan Cui,[b] Muhammad Asad Ziaee,[b] Yaqiong Gong,[a] Rongjian Sa,[*c] Hong Zhong[*a,b]

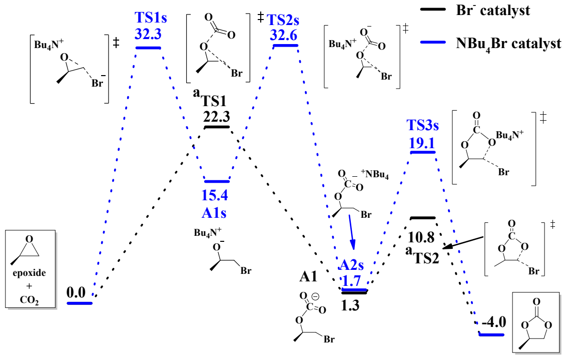

Figure S1. Free energy profiles for carbonate formation with bromine as catalyst.

Including nBu_4N^+ cation (shown in blue) the reaction energies is larger than the reaction without nBu_4N^+ cation included (shown in black). Then the nBu_4N^+ can be reduced eliminating in the further calculations.

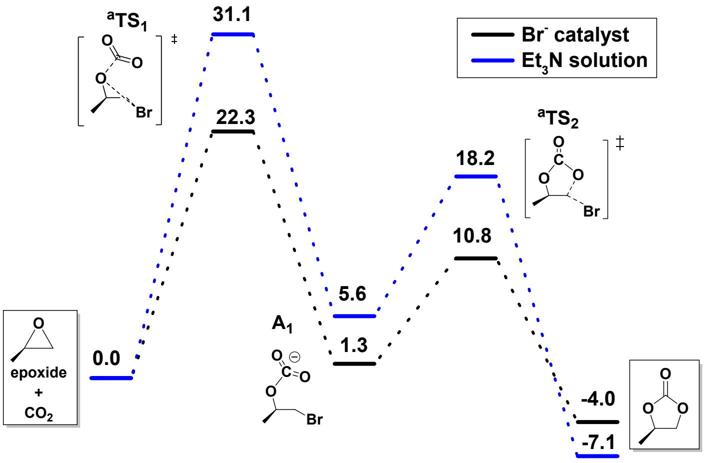

Figure S2. Free energy profiles with (shown in blue) or without (shown in black) triethylamine as solvent.

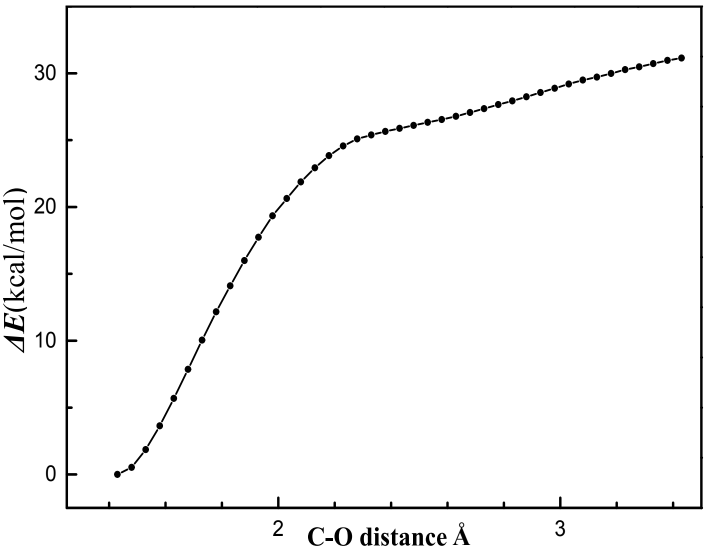

FigureS3. Potential Energy Surfaces scan along the distances between C atom of CO_2 moiety and O atom of epoxide moiety with one step set to 0.05 Å

No further transition states obtained in the formation of C1 intermediate after a potential energy surfaces scan calculation.

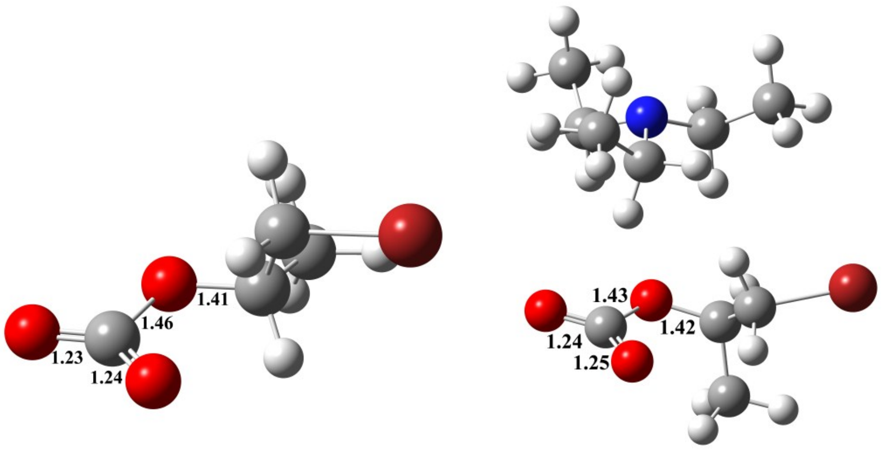

Figure S4. The bond length of A_1 and C_1 intermediate (only the bond around CO_2 moiety shown) in Å.

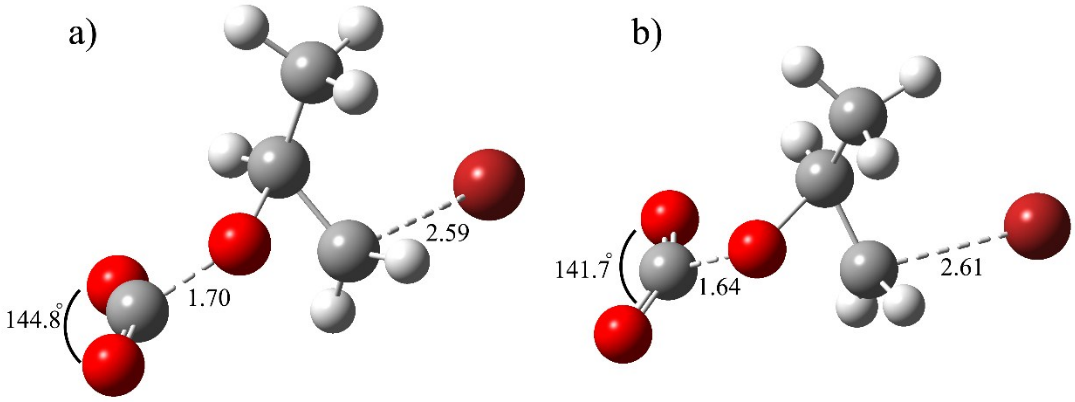

Figure S5. The optimized structure difference of ^{a}TS1in gas-phase (a) and TEA solvent (b).

^{1}H NMR spectrum

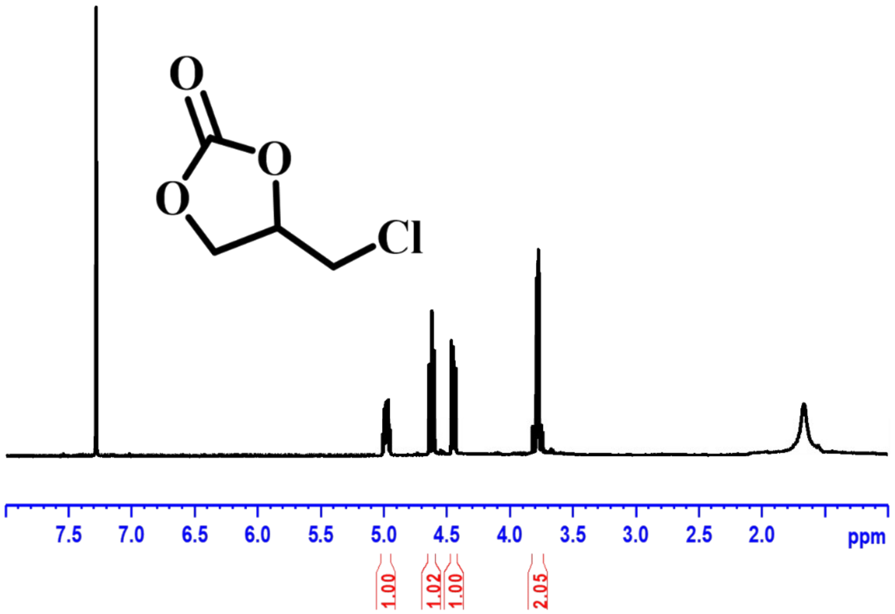

Figure S6. ^{1}**H NMR spectrum in chloroform-d**

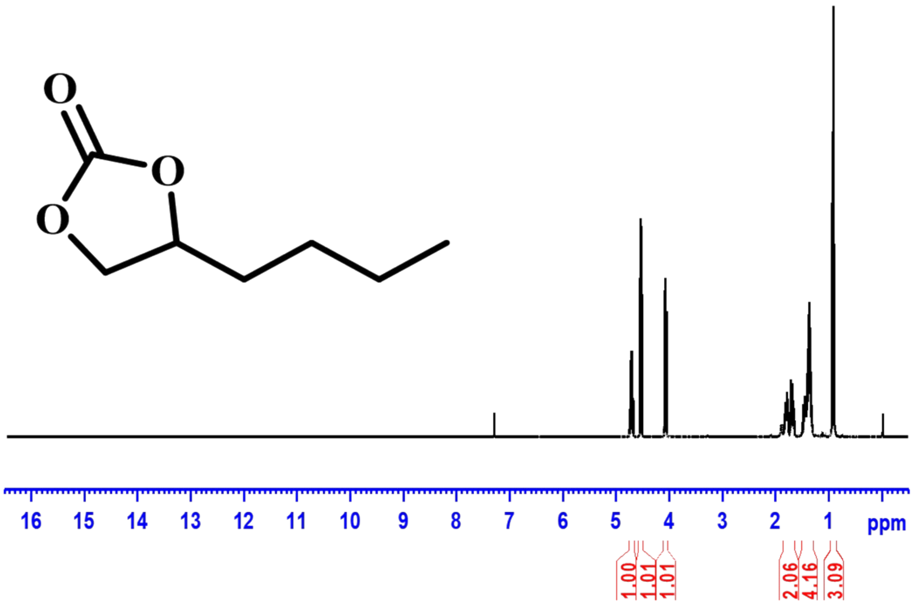

Figure S7. ^{1}**H NMR spectrum in chloroform-d**

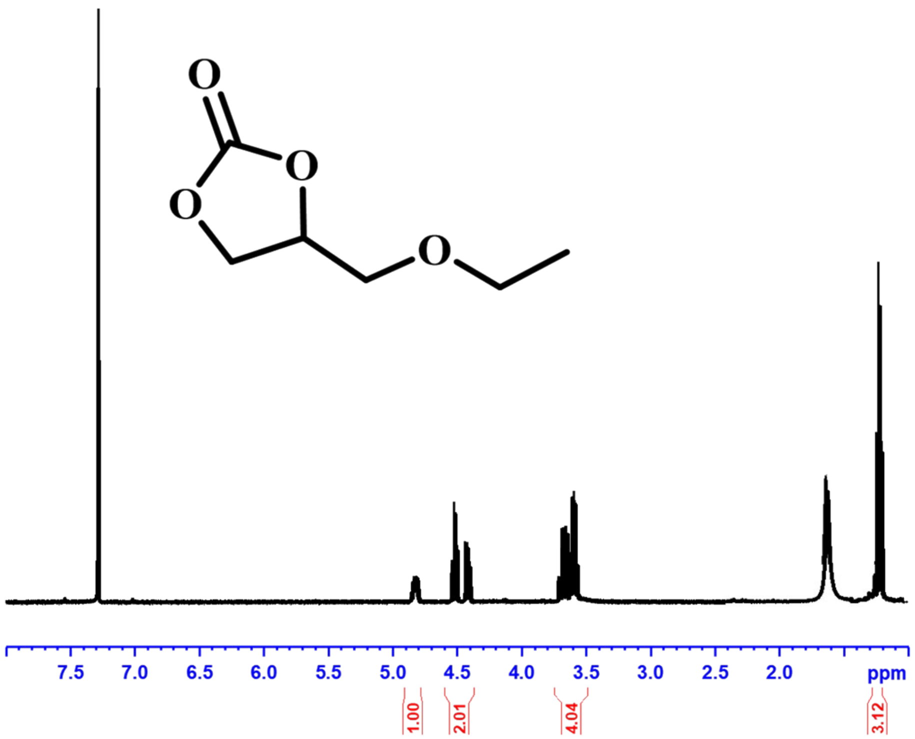

Figure S8. **^{1}H NMR spectrum in chloroform-d**

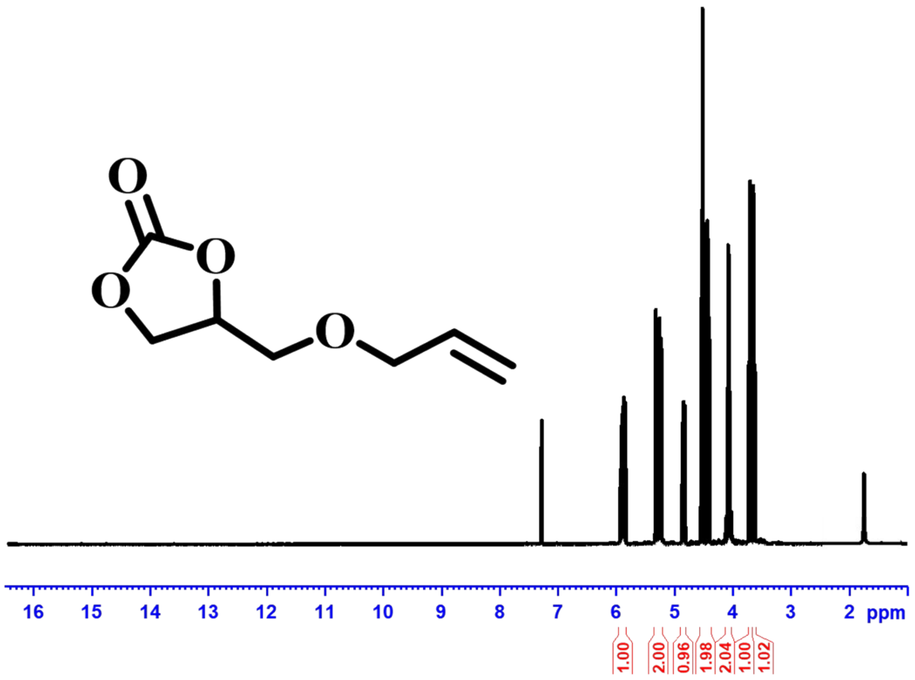

Figure S9. **^{1}H NMR spectrum in chloroform-d**

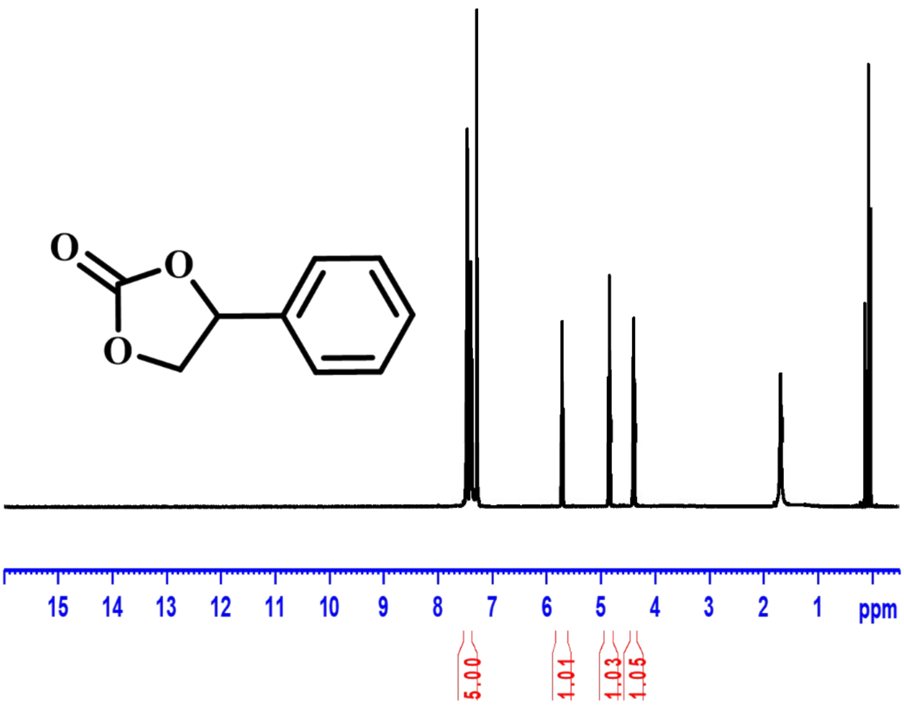

Figure S10. ^{1}**H NMR spectrum in chloroform-d**

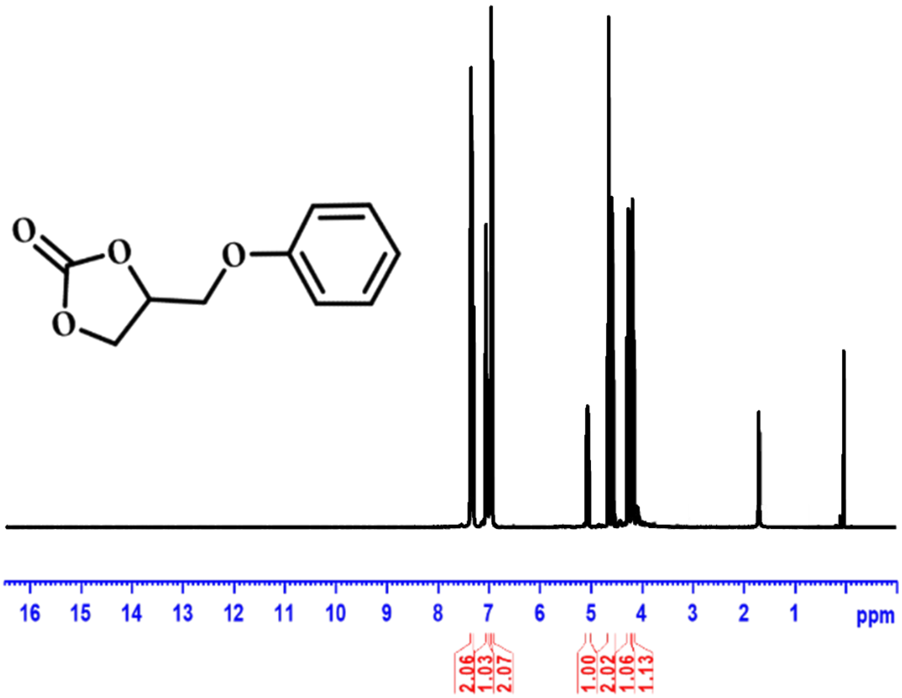

Figure S11. ^{1}**H NMR spectrum in chloroform-d**

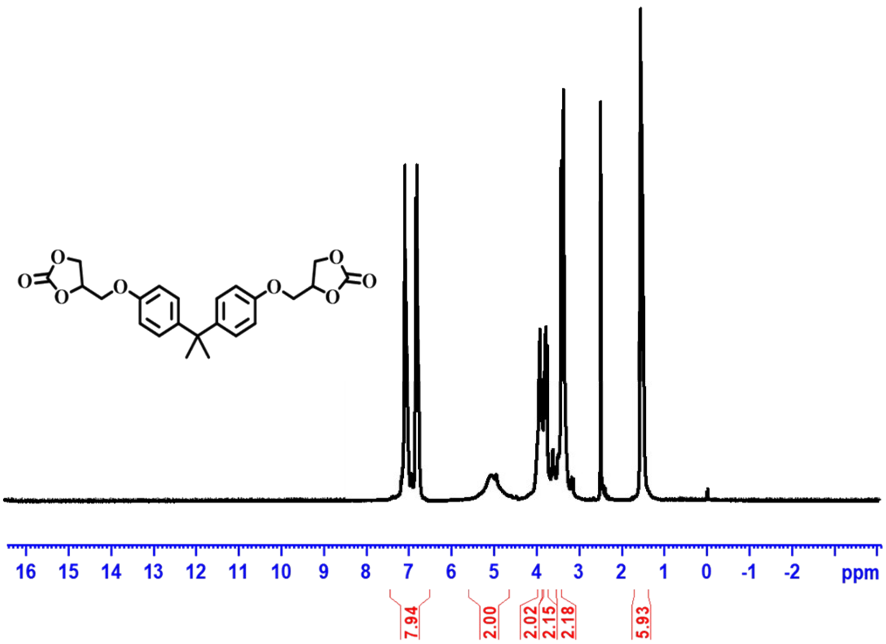

Figure S12. ^{1}**H NMR spectrum in dmso-d6**

Cartesian Coordinates and Energies.

Epoxide

Cartesian coordinates and thermal corrections at M06-2X/6-31+G(d,p) in gas phase

C	0.752861	-0.236626	-0.000424
C	2.222468	-0.253394	0.014856
N	1.502886	0.971129	-0.023744
H	0.214206	-0.464139	-0.917433
H	0.215548	-0.426790	0.927170
H	2.729416	-0.487960	-0.921626
C	2.996253	-0.528517	1.274874
H	3.265938	-1.586852	1.338565
H	3.915817	0.063100	1.295792
H	2.396332	-0.262741	2.149078

Zero-point correction=	0.086529 (Hartree/Particle)
Thermal correction to Energy=	0.090879
Thermal correction to Enthalpy=	0.091824
Thermal correction to Gibbs Free Energy=	0.060211
Sum of electronic and zero-point Energies=	-192.944191
Sum of electronic and thermal Energies=	-192.939841
Sum of electronic and thermal Enthalpies=	-192.938896
Sum of electronic and thermal Free Energies=	-192.970509

SCF-energy at M06-2X/6-311+G(d,p)

E_{scf}= -193.0768305 au

Cartesian coordinates and thermal corrections at M06-2X/6-31+G(d,p) in TEA solvent.

C	0.753671	-0.237962	-0.000025
C	2.221508	-0.253683	0.016104
N	1.501666	0.975439	-0.027245
H	0.215740	-0.467471	-0.917515
H	0.213267	-0.423804	0.926955
H	2.729835	-0.488754	-0.919464
C	2.995090	-0.526624	1.274676
H	3.265447	-1.585671	1.334190
H	3.917216	0.062336	1.295974
H	2.398285	-0.266594	2.153456

Zero-point correction=	0.086478 (Hartree/Particle)
Thermal correction to Energy=	0.090824
Thermal correction to Enthalpy=	0.091768
Thermal correction to Gibbs Free Energy=	0.060169
Sum of electronic and zero-point Energies=	-192.949479
Sum of electronic and thermal Energies=	-192.945133
Sum of electronic and thermal Enthalpies=	-192.944189
Sum of electronic and thermal Free Energies=	-192.975787

SCF-energy at M06-2X/6-311+G(d,p)
E_{scf}= -193.0820879au

CO_2

Cartesian coordinates and thermal corrections at M06-2X/6-31+G(d,p) in gas phase

C	0.402264	-1.135057	-0.005266
O	-0.760808	-1.135057	-0.005266
O	1.565336	-1.135057	-0.005266

Zero-point correction=	0.011927 (Hartree/Particle)
Thermal correction to Energy=	0.014539
Thermal correction to Enthalpy=	0.015484
Thermal correction to Gibbs Free Energy=	-0.009421
Sum of electronic and zero-point Energies=	-188.504125
Sum of electronic and thermal Energies=	-188.501513
Sum of electronic and thermal Enthalpies=	-188.500569
Sum of electronic and thermal Free Energies=	-188.525474

SCF-energy at M06-2X/6-311+G(d,p)
E_{scf}= -188.5745957au

Cartesian coordinates and thermal corrections at M06-2X/6-31+G(d,p) in TEA solvent

C	0.402264	-1.135057	-0.005266

O	-0.760480	-1.135057	-0.005266
O	1.565009	-1.135057	-0.005266

Zero-point correction= 0.011819 (Hartree/Particle)
Thermal correction to Energy= 0.014450
Thermal correction to Enthalpy= 0.015394
Thermal correction to Gibbs Free Energy= -0.008882
Sum of electronic and zero-point Energies= -188.506181
Sum of electronic and thermal Energies= -188.503550
Sum of electronic and thermal Enthalpies= -188.502606
Sum of electronic and thermal Free Energies= -188.526882

SCF-energy at M06-2X/6-311+G(d,p)
E_{scf}= -188.5765689au

Carbonate

Cartesian coordinates and thermal corrections at M06-2X/6-31+G(d,p) in gas phase

C	-0.492268	1.287271	-0.064822
C	-1.008063	-0.057180	0.464496
O	0.923119	1.109332	-0.050793
H	-0.813379	1.489568	-1.090884
H	-0.744475	2.130152	0.580587
C	1.209668	-0.215261	-0.046089
O	0.075518	-0.934441	0.123949
O	2.300401	-0.675753	-0.172148
C	-1.235131	-0.079507	1.964317
H	-1.425616	-1.099917	2.303089
H	-0.352926	0.303648	2.487179
H	-2.094847	0.544685	2.224706
H	-1.887830	-0.405692	-0.080033

Zero-point correction= 0.104316 (Hartree/Particle)
Thermal correction to Energy= 0.110418
Thermal correction to Enthalpy= 0.111362
Thermal correction to Gibbs Free Energy= 0.074134
Sum of electronic and zero-point Energies= -381.477865
Sum of electronic and thermal Energies= -381.471763
Sum of electronic and thermal Enthalpies= -381.470819
Sum of electronic and thermal Free Energies= -381.508048

SCF-energy at M06-2X/6-311+G(d,p)
E_{scf}= -381.6811057au

Cartesian coordinates and thermal corrections at M06-2X/6-31+G(d,p) in TEA solvent.

C	-0.496473	1.286799	-0.065753
C	-1.010261	-0.054374	0.466143
O	0.925754	1.107459	-0.055574

H	-0.815397	1.486123	-1.092572
H	-0.745205	2.132575	0.576826
C	1.205692	-0.211087	-0.044120
O	0.081624	-0.933146	0.123120
O	2.301977	-0.672968	-0.167785
C	-1.232937	-0.081088	1.963991
H	-1.436124	-1.100309	2.301107
H	-0.348474	0.292638	2.491200
H	-2.087965	0.549027	2.226240
H	-1.888039	-0.404745	-0.079272

Zero-point correction= 0.104524 (Hartree/Particle)
Thermal correction to Energy= 0.110596
Thermal correction to Enthalpy= 0.111540
Thermal correction to Gibbs Free Energy= 0.074373
Sum of electronic and zero-point Energies= -381.489467
Sum of electronic and thermal Energies= -381.483395
Sum of electronic and thermal Enthalpies= -381.482451
Sum of electronic and thermal Free Energies= -381.519618

SCF-energy at M06-2X/6-311+G(d,p)
E_{scf}= -381.693002au

Br^-

Thermal corrections at M06-2X/6-31+G(d,p) in gas phase

Zero-point correction= 0.000000 (Hartree/Particle)
Thermal correction to Energy= 0.001416
Thermal correction to Enthalpy= 0.002360
Thermal correction to Gibbs Free Energy= -0.016176
Sum of electronic and zero-point Energies= -2571.868813
Sum of electronic and thermal Energies= -2571.867396
Sum of electronic and thermal Enthalpies= -2571.866452
Sum of electronic and thermal Free Energies= -2571.884988

SCF-energy at M06-2X/6-311+G(d,p)
E_{scf}= -2574.2563064au

Thermal corrections at M06-2X/6-31+G(d,p) in TEA solvent

Zero-point correction= 0.000000 (Hartree/Particle)
Thermal correction to Energy= 0.001416
Thermal correction to Enthalpy= 0.002360
Thermal correction to Gibbs Free Energy= -0.016176
Sum of electronic and zero-point Energies= -2571.921767
Sum of electronic and thermal Energies= -2571.920351
Sum of electronic and thermal Enthalpies= -2571.919407

Sum of electronic and thermal Free Energies= -2571.937943

SCF-energy at M06-2X/6-311+G(d,p)

E$_{scf}$= -2574.3096898au

Trimethylamine

Cartesian coordinates and thermal corrections at M06-2X/6-31+G(d,p) in TEA solvent

N	-0.003700	0.000310	-0.012683
C	-1.315118	0.485564	0.421692
C	1.062795	0.891633	0.448789
C	-2.457246	-0.036594	-0.439726
C	1.210500	2.130587	-0.424807
C	0.224682	-1.375484	0.432411
C	1.276908	-2.098301	-0.398299
H	-1.309662	1.578028	0.360927
H	-1.488857	0.232773	1.485724
H	2.006413	0.338524	0.426707
H	0.895337	1.181500	1.504148
H	-3.410579	0.378900	-0.097651
H	-2.543043	-1.126608	-0.402298
H	-2.304681	0.254618	-1.483149
H	2.016715	2.769344	-0.049721
H	0.296693	2.732082	-0.447384
H	1.447160	1.839999	-1.452624
H	-0.718787	-1.921978	0.343886
H	0.499899	-1.396629	1.504910
H	1.388763	-3.131115	-0.052959
H	2.259246	-1.621562	-0.330512
H	0.979744	-2.112986	-1.451021

Zero-point correction= 0.206956 (Hartree/Particle)
Thermal correction to Energy= 0.216416
Thermal correction to Enthalpy= 0.217360
Thermal correction to Gibbs Free Energy= 0.172693
Sum of electronic and zero-point Energies= -292.076719
Sum of electronic and thermal Energies= -292.067260
Sum of electronic and thermal Enthalpies= -292.066316
Sum of electronic and thermal Free Energies= -292.110983

SCF-energy at M06-2X/6-311+G(d,p)

E$_{scf}$= -292.346056au

[a]TS1

Cartesian coordinates and thermal corrections at M06-2X/6-31+G(d,p) in gas phase

C	-0.58393	0.81098	-0.33167
C	0.06616	-0.08376	0.63993
O	-1.74894	0.60479	0.36463
H	0.23122	0.32591	1.62917
H	-0.19219	-1.13159	0.58243
Br	2.33813	-0.44544	0.14867
H	-0.56583	0.39437	-1.35812
C	-0.07908	2.24536	-0.34756
H	-0.03179	2.62637	0.67928
H	0.91668	2.31807	-0.79899
H	-0.77519	2.8758	-0.91237

Zero-point correction= 0.084871 (Hartree/Particle)
Thermal correction to Energy= 0.090857
Thermal correction to Enthalpy= 0.091801
Thermal correction to Gibbs Free Energy= 0.053888
Sum of electronic and zero-point Energies= -2764.792760
Sum of electronic and thermal Energies= -2764.786775
Sum of electronic and thermal Enthalpies= -2764.785830
Sum of electronic and thermal Free Energies= -2764.823744
Imaginary frequency: 427.4129 cm^{-1}
IR Intensity: 1059.9617

SCF-energy at M06-2X/6-311+G(d,p)
$\mathbf{E_{scf}}$= -2767.3045104au

Cartesian coordinates and thermal corrections at M06-2X/6-31+G(d,p) in TEA solvent

C	1.114602	0.741141	-0.655378
C	0.325870	-0.501343	-0.712713
O	2.201813	-0.014897	-1.014868
H	0.116877	-0.887944	-1.702777
H	0.514077	-1.229286	0.063854
Br	-1.920687	-0.182848	-0.101416
H	0.719070	1.826212	-1.645412
C	1.514485	2.578240	-1.702877
H	0.594280	1.386735	-2.642080
H	-0.216459	2.317515	-1.355439
H	1.151123	1.164114	0.368172

Zero-point correction= 0.084841 (Hartree/Particle)
Thermal correction to Energy= 0.090887
Thermal correction to Enthalpy= 0.091831
Thermal correction to Gibbs Free Energy= 0.053730
Sum of electronic and zero-point Energies= -2764.841737
Sum of electronic and thermal Energies= -2764.835691

Sum of electronic and thermal Enthalpies= -2764.834747
Sum of electronic and thermal Free Energies= -2764.872847
Imaginary frequency: 478.2540 cm^{-1}
IR Intensity: 1433.0586

SCF-energy at M06-2X/6-311+G(d,p)
Escf= -2767.353739au

A1

Cartesian coordinates and thermal corrections at M06-2X/6-31+G(d,p) in gas phase

C	1.671069	0.503030	-0.160313
C	0.677193	-0.665231	-0.247972
O	2.851950	-0.049471	-0.474566
H	0.560416	-1.007985	-1.277374
H	0.991045	-1.476964	0.404758
Br	-1.229740	-0.277994	0.372791
C	1.242016	1.640617	-1.110104
H	1.998373	2.431676	-1.068960
H	1.218815	1.250541	-2.136266
H	0.259498	2.062856	-0.859788
H	1.592950	0.908635	0.885191

Zero-point correction= 0.085084 (Hartree/Particle)
Thermal correction to Energy= 0.091256
Thermal correction to Enthalpy= 0.092200
Thermal correction to Gibbs Free Energy= 0.054065
Sum of electronic and zero-point Energies= -2764.797088
Sum of electronic and thermal Energies= -2764.790916
Sum of electronic and thermal Enthalpies= -2764.789972
Sum of electronic and thermal Free Energies= -2764.828107

SCF-energy at M06-2X/6-311+G(d,p)
$\mathbf{E_{scf}}$= -2767.3072016au

Cartesian coordinates and thermal corrections at M06-2X/6-31+G(d,p) in TEA solvent

C	1.667878	0.497483	-0.161324
C	0.666941	-0.665942	-0.249047
O	2.867563	-0.033867	-0.460148
H	0.547024	-1.008908	-1.278868
H	0.987732	-1.485567	0.391876
Br	-1.202017	-0.260825	0.386388
C	1.243044	1.633317	-1.110822
H	1.990326	2.433033	-1.062907
H	1.225518	1.253507	-2.141274

H	0.257851	2.053561	-0.868944
H	1.581724	0.903916	0.882470

Zero-point correction= 0.085463 (Hartree/Particle)
Thermal correction to Energy= 0.091559
Thermal correction to Enthalpy= 0.092504
Thermal correction to Gibbs Free Energy= 0.054527
Sum of electronic and zero-point Energies= -2764.849378
Sum of electronic and thermal Energies= -2764.843282
Sum of electronic and thermal Enthalpies= -2764.842338
Sum of electronic and thermal Free Energies= -2764.880314

SCF-energy at M06-2X/6-311+G(d,p)
$\mathbf{E_{scf}}$= -2767.3598537au

[a]TS$_2$

Cartesian coordinates and thermal corrections at M06-2X/6-31+G(d,p) in gas phase

C	-1.234806	0.981867	-0.148252
C	-0.687402	0.145398	0.899504
O	-2.341564	0.683806	0.714825
H	-0.411402	0.587609	1.845036
H	-0.739655	-0.928932	0.797218
Br	1.842148	0.035463	0.373937
C	-0.870531	2.440533	-0.192294
H	-0.868922	2.855897	0.819562
H	0.134080	2.540407	-0.612439
H	-1.586616	2.997575	-0.803600
H	-1.331478	0.492878	-1.117577
C	-3.460133	-0.483817	0.174932
O	-3.065677	-0.965188	-0.858739
O	-4.338833	-0.499047	0.992577

Zero-point correction= 0.099256 (Hartree/Particle)
Thermal correction to Energy= 0.108574
Thermal correction to Enthalpy= 0.109518
Thermal correction to Gibbs Free Energy= 0.062238
Sum of electronic and zero-point Energies= -2953.318462
Sum of electronic and thermal Energies= -2953.309144
Sum of electronic and thermal Enthalpies= -2953.308200
Sum of electronic and thermal Free Energies= -2953.355480
Imaginary frequency: 523.9255 cm^{-1}
IR Intensity: 1860.0924

SCF-energy at M06-2X/6-311+G(d,p)
$\mathbf{E_{scf}}$= -2955.8997816au

Cartesian coordinates and thermal corrections at M06-2X/6-31+G(d,p) in TEA solvent

C	-0.505298	0.724207	-0.281429
C	0.338453	-0.152633	0.636700
O	-1.825195	0.705928	0.222247
C	-2.615304	-0.463903	-0.049893
O	-2.063760	-1.348509	-0.730736
O	-3.742788	-0.392470	0.452674
H	0.435936	0.291338	1.628816
H	-0.078658	-1.155763	0.691694
Br	2.166486	-0.369997	-0.039409
H	-0.477263	0.303229	-1.292177
C	-0.044868	2.171693	-0.279280
H	-0.100754	2.584618	0.734239
H	0.984734	2.258103	-0.638370
H	-0.692767	2.770304	-0.925492

Zero-point correction= 0.102553 (Hartree/Particle)
Thermal correction to Energy= 0.111112
Thermal correction to Enthalpy= 0.112056
Thermal correction to Gibbs Free Energy= 0.066906
Sum of electronic and zero-point Energies= -2953.412090
Sum of electronic and thermal Energies= -2953.403531
Sum of electronic and thermal Enthalpies= -2953.402587
Sum of electronic and thermal Free Energies= -2953.447737
Imaginary frequency: 534.5117 cm^{-1}
IR Intensity: 2236.1393

SCF-energy at M06-2X/6-311+G(d,p)
$\mathbf{E_{scf}}$= -2955.946891au

^{b}TS$_1$

Cartesian coordinates and thermal corrections at M06-2X/6-31+G(d,p) in TEA solvent

C	0.681289	-1.315266	-1.388473
C	1.891422	-1.383830	-0.516921
O	1.569162	-0.101841	-0.173619
H	0.764444	-0.610598	-2.206522
H	-0.258191	-1.320336	-0.844023
H	2.834433	-1.493757	-1.087610
C	1.903433	-2.351691	0.659212
H	2.113693	-3.390275	0.376899
H	2.686310	-2.023111	1.351093
H	0.950332	-2.305275	1.197049
N	0.185820	-2.771661	-2.586413
C	-1.290336	-2.755277	-2.729709

C	0.641950	-4.068074	-2.033593
C	-1.855412	-1.507116	-3.395754
C	-0.148530	-4.554848	-0.827072
C	0.824743	-2.521573	-3.899957
C	2.341371	-2.634119	-3.905424
H	-1.712574	-2.843138	-1.725951
H	-1.596534	-3.650462	-3.294374
H	1.692681	-3.943211	-1.761381
H	0.593496	-4.824735	-2.833311
H	-2.944469	-1.530840	-3.300191
H	-1.623817	-1.457307	-4.462122
H	-1.502546	-0.590015	-2.915858
H	0.400838	-5.373351	-0.353277
H	-1.132634	-4.939442	-1.105907
H	-0.279363	-3.769150	-0.078953
H	0.546251	-1.511343	-4.209558
H	0.393535	-3.222596	-4.631950
H	2.706193	-2.326144	-4.889268
H	2.690797	-3.654463	-3.730963
H	2.796147	-1.975403	-3.161553

Zero-point correction= 0.296439 (Hartree/Particle)
Thermal correction to Energy= 0.309984
Thermal correction to Enthalpy= 0.310929
Thermal correction to Gibbs Free Energy= 0.257492
Sum of electronic and zero-point Energies= -484.979341
Sum of electronic and thermal Energies= -484.965795
Sum of electronic and thermal Enthalpies= -484.964851
Sum of electronic and thermal Free Energies= -485.018287
Imaginary frequency: 582.7656 cm^{-1}
IR Intensity: 1394.1001

SCF-energy at M06-2X/6-311+G(d,p)
$\mathbf{E_{scf}}$= -485.3851638au

B1

Cartesian coordinates and thermal corrections at M06-2X/6-31+G(d,p) in TEA solvent

C	0.31553	-1.6058	-1.28793
C	1.68457	-1.1239	-0.69086
O	1.33435	-0.11475	0.11505
H	-0.12589	-0.70776	-1.71858
H	-0.30574	-1.922	-0.44711
H	2.32677	-0.8118	-1.55636
C	2.44891	-2.23383	0.0635

H	2.91474	-3.00617	-0.56385
H	3.24759	-1.74078	0.62557
H	1.78646	-2.71635	0.79432
N	0.14011	-2.68751	-2.37226
C	-1.31626	-2.68781	-2.79869
C	0.47228	-4.06993	-1.86264
C	-1.77126	-1.4953	-3.624
C	-0.44558	-4.61096	-0.779
C	0.99011	-2.38519	-3.58732
C	2.44548	-2.81886	-3.52306
H	-1.89933	-2.74429	-1.87826
H	-1.46805	-3.6146	-3.36323
H	1.49351	-4.02689	-1.49119
H	0.4513	-4.72642	-2.73937
H	-2.84317	-1.61967	-3.80183
H	-1.28347	-1.44167	-4.59911
H	-1.63719	-0.54391	-3.10598
H	-0.02843	-5.56747	-0.45164
H	-1.45987	-4.80305	-1.1352
H	-0.49026	-3.96185	0.09806
H	0.92052	-1.30564	-3.73691
H	0.50172	-2.88352	-4.43077
H	2.9345	-2.42376	-4.41824
H	2.56117	-3.90482	-3.54133
H	2.96887	-2.41398	-2.65722

Zero-point correction= 0.298825 (Hartree/Particle)
Thermal correction to Energy= 0.312271
Thermal correction to Enthalpy= 0.313216
Thermal correction to Gibbs Free Energy= 0.260497
Sum of electronic and zero-point Energies= -484.993879
Sum of electronic and thermal Energies= -484.980432
Sum of electronic and thermal Enthalpies= -484.979488
Sum of electronic and thermal Free Energies= -485.032206

SCF-energy at M06-2X/6-311+G(d,p)
E_{scf}= -485.4021185au

$^{b}TS_2$

Cartesian coordinates and thermal corrections at M06-2X/6-31+G(d,p) in TEA solvent

C	0.558930	-1.053562	-1.546784
C	1.756320	-1.012271	-0.668298
O	1.306204	0.260695	-0.395281
H	0.606188	-0.409536	-2.415682

H	-0.392450	-1.124554	-1.028875
H	2.709933	-1.045252	-1.222783
C	1.828846	-1.909365	0.555100
H	2.127833	-2.936687	0.319505
H	2.572621	-1.487227	1.238828
H	0.867468	-1.920468	1.078864
N	0.208256	-2.629098	-2.701867
C	-1.259843	-2.739830	-2.868609
C	0.760590	-3.854498	-2.082463
C	-1.907517	-1.572511	-3.603306
C	-0.004993	-4.347143	-0.862214
C	0.852709	-2.379763	-4.011941
C	2.373523	-2.354790	-3.982279
H	-1.693824	-2.812426	-1.868388
H	-1.486026	-3.681742	-3.394392
H	1.793914	-3.631786	-1.805174
H	0.787554	-4.651413	-2.843650
H	-2.993119	-1.668403	-3.514438
H	-1.668115	-1.561015	-4.669004
H	-1.626088	-0.608516	-3.170379
H	0.598965	-5.100273	-0.348128
H	-0.953778	-4.817267	-1.131968
H	-0.204975	-3.541752	-0.151104
H	0.492692	-1.413079	-4.373124
H	0.502410	-3.146123	-4.722168
H	2.732711	-2.040641	-4.966285
H	2.807836	-3.335435	-3.773155
H	2.750467	-1.636979	-3.249535
C	2.191958	1.890784	-1.904084
O	1.706378	2.812617	-1.383753
O	2.753540	1.114191	-2.576552

Zero-point correction= 0.310088 (Hartree/Particle)
Thermal correction to Energy= 0.327281
Thermal correction to Enthalpy= 0.328225
Thermal correction to Gibbs Free Energy= 0.265808
Sum of electronic and zero-point Energies= -673.495775
Sum of electronic and thermal Energies= -673.478582
Sum of electronic and thermal Enthalpies= -673.477638
Sum of electronic and thermal Free Energies= -673.540055
Imaginary frequency: 581.4140 cm^{-1}
IR Intensity: 1462.6471
SCF-energy at M06-2X/6-311+G(d,p)
$\mathbf{E_{scf}}$= -673.9732952au

B2

Cartesian coordinates and thermal corrections at M06-2X/6-31+G(d,p) in TEA solvent

C	-0.568761	-0.100307	0.388564
C	0.786745	0.174844	1.077748
O	0.882410	1.566899	1.274490
H	-0.839570	0.795305	-0.165939
H	-1.338667	-0.298558	1.140234
H	1.608498	-0.166249	0.443145
C	0.903728	-0.457897	2.457496
H	0.922820	-1.551210	2.414015
H	1.831732	-0.121488	2.926089
H	0.070511	-0.147092	3.096773
N	-0.646558	-1.236320	-0.616174
C	-2.111928	-1.460409	-0.950372
C	-0.089601	-2.523189	-0.058246
C	-2.795067	-0.315463	-1.677938
C	-0.893960	-3.136607	1.077643
C	0.065388	-0.898406	-1.930892
C	1.561120	-1.142635	-2.014833
H	-2.619465	-1.655330	-0.004795
H	-2.139950	-2.374429	-1.551894
H	0.930598	-2.303857	0.258652
H	-0.034277	-3.218241	-0.901152
H	-3.835946	-0.610418	-1.837379
H	-2.357597	-0.111929	-2.656498
H	-2.804488	0.608066	-1.095596
H	-0.281480	-3.920637	1.531293
H	-1.816859	-3.604114	0.729055
H	-1.136391	-2.418325	1.864866
H	-0.134546	0.156752	-2.116168
H	-0.443707	-1.511820	-2.680464
H	1.839387	-0.917174	-3.049035
H	1.845800	-2.179777	-1.822853
H	2.125826	-0.456838	-1.386617
C	1.130570	2.362501	0.101104
O	1.193449	3.564926	0.343667
O	1.231153	1.713402	-0.963804

Zero-point correction= 0.317100 (Hartree/Particle)
Thermal correction to Energy= 0.332650
Thermal correction to Enthalpy= 0.333594
Thermal correction to Gibbs Free Energy= 0.275732
Sum of electronic and zero-point Energies= -673.551599
Sum of electronic and thermal Energies= -673.536049

Sum of electronic and thermal Enthalpies= -673.535105
Sum of electronic and thermal Free Energies= -673.592966

SCF-energy at M06-2X/6-311+G(d,p)
E_{scf}= -674.0323537au

$^{b}TS_3$

Cartesian coordinates and thermal corrections at M06-2X/6-31+G(d,p) in TEA solvent

C	0.502619	-0.631743	-2.052254
C	1.702703	-0.427535	-1.115071
O	1.244154	0.414631	-0.062890
H	0.593809	-0.356655	-3.090629
H	-0.479429	-0.728678	-1.610157
H	2.449383	0.125176	-1.702513
C	2.397560	-1.584928	-0.420343
H	2.935758	-2.222598	-1.121003
H	3.132225	-1.147027	0.260170
H	1.707768	-2.182200	0.177953
N	0.286402	-2.522697	-2.739562
C	-1.161863	-2.535419	-3.113841
C	0.514301	-3.667977	-1.821627
C	-1.602234	-1.586070	-4.221684
C	-0.355439	-3.638230	-0.569822
C	1.108281	-2.666099	-3.965483
C	2.612287	-2.648852	-3.750590
H	-1.727574	-2.303668	-2.208227
H	-1.419368	-3.566132	-3.400348
H	1.564977	-3.678536	-1.543950
H	0.320239	-4.594418	-2.385236
H	-2.691562	-1.652520	-4.293938
H	-1.199634	-1.857654	-5.199863
H	-1.359829	-0.541231	-4.014649
H	0.059387	-4.329816	0.168676
H	-1.382606	-3.952205	-0.769112
H	-0.384807	-2.643802	-0.113784
H	0.847308	-1.837619	-4.628137
H	0.807694	-3.594732	-4.474913
H	3.097323	-2.726617	-4.727823
H	2.971146	-3.483817	-3.143849
H	2.941718	-1.709716	-3.297263
C	0.499996	1.484741	-0.561630
O	0.213935	2.385126	0.206543
O	0.214773	1.329249	-1.797244

Zero-point correction= 0.314413 (Hartree/Particle)
Thermal correction to Energy= 0.329856
Thermal correction to Enthalpy= 0.330800
Thermal correction to Gibbs Free Energy= 0.272815
Sum of electronic and zero-point Energies= -673.504943
Sum of electronic and thermal Energies= -673.489501
Sum of electronic and thermal Enthalpies= -673.488557
Sum of electronic and thermal Free Energies= -673.546542
Imaginary frequency: 581.9400 cm^{-1}
IR Intensity: 1485.1047

SCF-energy at M06-2X/6-311+G(d,p)
$\mathbf{E_{scf}}$= -673.9822709au

C1

Cartesian coordinates and thermal corrections at M06-2X/6-31+G(d,p) in TEA solvent

N	0.684809	-1.350674	-2.561125
C	-0.770899	-1.277517	-2.715564
C	1.063772	-2.564849	-1.830722
C	-1.265294	-0.054880	-3.479682
C	0.410373	-2.698428	-0.459715
C	1.351831	-1.293316	-3.865646
C	2.869427	-1.419010	-3.798377
H	-1.209327	-1.251098	-1.714563
H	-1.149166	-2.193821	-3.212908
H	2.147113	-2.542370	-1.688580
H	0.835202	-3.464333	-2.438065
H	-2.353025	0.011858	-3.383723
H	-1.034407	-0.096864	-4.546967
H	-0.841148	0.870452	-3.074614
H	0.909406	-3.494678	0.100620
H	-0.649509	-2.959340	-0.517606
H	0.508435	-1.767937	0.107604
H	1.112486	-0.326793	-4.317099
H	0.947720	-2.077489	-4.537554
H	3.296120	-1.136202	-4.765295
H	3.201245	-2.437673	-3.581485
H	3.282562	-0.745621	-3.040764
C	1.292878	1.035358	-1.357500
O	0.683223	0.793994	-0.394961
O	1.928554	1.384008	-2.269053

Zero-point correction= 0.311568 (Hartree/Particle)
Thermal correction to Energy= 0.331092

Thermal correction to Enthalpy= 0.332036
Thermal correction to Gibbs Free Energy= 0.261428
Sum of electronic and zero-point Energies= -3245.494303
Sum of electronic and thermal Energies= -3245.474779
Sum of electronic and thermal Enthalpies= -3245.473835
Sum of electronic and thermal Free Energies= -3245.544443

SCF-energy at M06-2X/6-311+G(d,p)
$\mathbf{E_{scf}}$= -480.9313697au

^{c}TS$_1$

Cartesian coordinates and thermal corrections at M06-2X/6-31+G(d,p) in TEA solvent

C	2.750384	1.021644	-0.754849
C	2.049807	-0.330696	-0.724495
O	1.782051	2.064436	-0.655499
H	3.376603	1.130618	0.131588
C	3.585312	1.178898	-2.015984
C	0.588418	1.757760	-1.293640
O	0.524448	0.542665	-1.674902
O	-0.237929	2.654250	-1.405147
H	1.354405	-0.571554	0.063553
H	2.099696	-0.998406	-1.570145
Br	3.720776	-1.585577	0.346381
H	2.947440	1.096893	-2.902287
H	4.072691	2.157502	-2.023503
H	4.354752	0.401908	-2.054834
N	0.087316	0.751963	2.977752
C	-0.346055	2.140674	2.792064
C	-0.530552	-0.122601	1.976202
C	-0.259685	2.953364	4.077781
C	-1.960473	-0.500706	2.340565
C	1.550403	0.653280	2.944157
C	2.076618	-0.603354	3.623038
H	-1.387503	2.132605	2.456323
H	0.232790	2.625584	1.985787
H	0.063384	-1.040526	1.910574
H	-0.505546	0.347777	0.975262
H	-0.601189	3.979273	3.904218
H	0.763163	3.008172	4.463866
H	-0.888045	2.502221	4.852453
H	-2.389331	-1.152956	1.572863
H	-2.607157	0.378190	2.424485
H	-1.978356	-1.028071	3.299475

H	1.956177	1.525658	3.466608
H	1.925315	0.715258	1.906155
H	3.170676	-0.606320	3.612932
H	1.755447	-1.516924	3.114908
H	1.727920	-0.639763	4.660826

Zero-point correction= 0.310907 (Hartree/Particle)
Thermal correction to Energy= 0.329931
Thermal correction to Enthalpy= 0.330875
Thermal correction to Gibbs Free Energy= 0.261380
Sum of electronic and zero-point Energies= -3245.477665
Sum of electronic and thermal Energies= -3245.458642
Sum of electronic and thermal Enthalpies= -3245.457697
Sum of electronic and thermal Free Energies= -3245.527193
Imaginary frequency: 505.6953 cm^{-1}
IR Intensity: 1406.6195

SCF-energy at M06-2X/6-311+G(d,p)
$\mathbf{E_{scf}}$= -3248.3237758au

nBu_4NBr

Cartesian coordinates and thermal corrections at M06-2X/6-31+G(d,p) in gas phase

C	-1.486681	1.796433	-0.818403
C	-2.625136	0.843442	-1.170931
C	-3.945638	1.615722	-1.218587
C	-5.107332	0.720387	-1.642854
C	-0.047975	0.715057	0.932642
C	-1.279779	0.082337	1.557501
C	-0.948616	-0.260722	3.013935
C	-2.017823	-1.166791	3.617222
C	0.112967	0.051356	-1.505690
C	1.542056	-0.443409	-1.634632
C	1.575161	-1.591048	-2.648143
C	2.934958	-2.283105	-2.636167
C	0.874870	2.303943	-0.726199
C	2.266237	2.138357	-0.089014
C	2.542563	3.174390	1.004143
C	3.944777	3.026615	1.587636
N	-0.131941	1.183484	-0.518650
Br	0.414151	-2.700956	0.622830
H	-1.741659	2.403463	0.056429
H	-1.326902	2.478258	-1.660947
H	-2.445865	0.392910	-2.152321
H	-2.706834	0.022142	-0.458142

H	-4.152179	2.044368	-0.229279
H	-3.855560	2.461208	-1.913266
H	-6.047702	1.276407	-1.668293
H	-4.934847	0.302310	-2.639444
H	-5.226873	-0.115121	-0.946537
H	0.229464	1.607957	1.503090
H	0.753565	-0.025861	0.977818
H	-2.148108	0.753525	1.535082
H	-1.514308	-0.852086	1.040593
H	0.014691	-0.782942	3.039127
H	-0.848537	0.664257	3.598705
H	-1.801453	-1.392445	4.664759
H	-3.008445	-0.699662	3.569563
H	-2.050482	-2.110663	3.064565
H	-0.512634	-0.784390	-1.176926
H	-0.238909	0.431248	-2.471853
H	2.215005	0.353290	-1.976274
H	1.893622	-0.837920	-0.677395
H	0.803531	-2.319193	-2.373884
H	1.343187	-1.209018	-3.652009
H	2.975163	-3.094333	-3.367876
H	3.742757	-1.578833	-2.867039
H	3.117251	-2.709030	-1.644615
H	0.395899	3.196228	-0.308736
H	0.938806	2.436402	-1.809764
H	2.402983	1.134658	0.318080
H	3.022075	2.243905	-0.874885
H	2.419051	4.182260	0.586697
H	1.798334	3.078327	1.804433
H	4.131564	3.774123	2.362443
H	4.079078	2.036709	2.034369
H	4.705908	3.148816	0.810488

Zero-point correction= 0.511061 (Hartree/Particle)
Thermal correction to Energy= 0.535333
Thermal correction to Enthalpy= 0.536277
Thermal correction to Gibbs Free Energy= 0.455256
Sum of electronic and zero-point Energies= -3257.116834
Sum of electronic and thermal Energies= -3257.092562
Sum of electronic and thermal Enthalpies= -3257.091618
Sum of electronic and thermal Free Energies= -3257.172639

SCF-energy at M06-2X/6-311+G(d,p)

$\mathbf{E_{scf}}$= -3260.1433287au

TS1s

Cartesian coordinates and thermal corrections at M06-2X/6-31+G(d,p) in gas phase

C	2.044434	1.158400	-0.867223
C	2.704481	1.922514	0.275780
C	3.446377	3.130300	-0.303622
C	4.046260	4.009344	0.790890
C	2.412391	-1.323002	-0.689369
C	3.715080	-1.197510	0.087197
C	4.550317	-2.468339	-0.092778
C	5.889670	-2.381477	0.634499
C	0.805054	-0.127545	0.837429
C	-0.120934	-1.252779	1.283196
C	-0.592711	-0.980672	2.716411
C	-1.627834	-2.007187	3.169628
C	0.352303	-0.379073	-1.630480
C	-0.257413	-1.772020	-1.825353
C	-1.731843	-1.647911	-2.228269
C	-2.355735	-3.003884	-2.541710
N	1.417377	-0.202681	-0.553226
H	2.752281	0.969061	-1.682610
H	1.212508	1.772991	-1.226988
H	1.929196	2.285469	0.957575
H	3.405477	1.313159	0.855225
H	4.240177	2.784397	-0.979187
H	2.747692	3.719590	-0.909382
H	4.578663	4.863474	0.364950
H	3.263275	4.395752	1.450174
H	4.755237	3.443875	1.404802
H	2.630823	-1.395906	-1.760912
H	1.892857	-2.238693	-0.397770
H	4.292498	-0.336743	-0.268621
H	3.525326	-1.045526	1.156129
H	3.982631	-3.330619	0.277908
H	4.721021	-2.643756	-1.162430
H	6.471117	-3.297024	0.502073
H	6.487576	-1.546148	0.256744
H	5.742569	-2.229576	1.708396
H	1.647726	-0.053330	1.528671
H	0.269310	0.824861	0.842536
H	-1.016288	-1.316361	0.655587
H	0.379493	-2.230098	1.251819
H	0.270676	-0.972561	3.395085
H	-1.045173	0.017013	2.757371

H	-1.927413	-1.826934	4.205227
H	-2.522663	-1.934365	2.543952
H	-1.229564	-3.026059	3.105173
H	0.841121	-0.064299	-2.558210
H	-0.391946	0.384257	-1.394954
H	0.294588	-2.314065	-2.603463
H	-0.196681	-2.385926	-0.925047
H	-2.298726	-1.166913	-1.418838
H	-1.815532	-0.986867	-3.100952
H	-3.407830	-2.892262	-2.813061
H	-1.838858	-3.499727	-3.370671
H	-2.307537	-3.663148	-1.668594
C	-2.103448	2.506256	-0.546940
C	-2.044103	1.364991	0.370121
O	-0.716621	2.430366	-0.385785
H	-1.683905	0.445460	-0.046970
H	-1.894472	1.559051	1.422163
Br	-4.029324	0.080714	0.759451
C	-2.640599	2.251550	-1.939497
H	-2.366409	3.076153	-2.604998
H	-2.222190	1.324654	-2.346688
H	-3.729701	2.149518	-1.916857
H	-2.551751	3.407271	-0.105737

Zero-point correction= 0.597773 (Hartree/Particle)
Thermal correction to Energy= 0.627684
Thermal correction to Enthalpy= 0.628628
Thermal correction to Gibbs Free Energy= 0.535015
Sum of electronic and zero-point Energies= -3450.030658
Sum of electronic and thermal Energies= -3450.000747
Sum of electronic and thermal Enthalpies= -3449.999802
Sum of electronic and thermal Free Energies= -3450.093416
Imaginary frequency: 479.5765 cm^{-1}
IR Intensity: 983.2199

SCF-energy at M06-2X/6-311+G(d,p)
$\mathbf{E_{scf}}$= -3453.1882547 au

A1s

Cartesian coordinates and thermal corrections at M06-2X/6-31+G(d,p) in gas phase

C	1.886283	1.113040	-1.087531
C	2.228553	2.267507	-0.154061
C	2.675850	3.464815	-0.996772
C	2.954545	4.694532	-0.135963

C	2.815877	-1.079155	-0.298539
C	3.984878	-0.449522	0.445881
C	5.126110	-1.461149	0.581569
C	6.341448	-0.861428	1.284984
C	0.871329	0.000271	0.872636
C	0.186333	-1.182672	1.537674
C	-0.292816	-0.752679	2.929132
C	-1.274475	-1.753161	3.533692
C	0.646913	-0.926224	-1.456255
C	0.495513	-2.442372	-1.391782
C	-0.673661	-2.851933	-2.293603
C	-0.836001	-4.367360	-2.376422
N	1.575048	-0.247656	-0.450734
H	2.694000	0.917179	-1.803272
H	0.963716	1.393674	-1.606217
H	1.323234	2.547733	0.393002
H	3.012402	2.022680	0.571251
H	3.576134	3.200804	-1.568287
H	1.890546	3.694125	-1.726770
H	3.271592	5.544090	-0.746473
H	2.056893	4.991879	0.414529
H	3.745165	4.491662	0.594526
H	3.131741	-1.336580	-1.316126
H	2.514135	-2.006339	0.196138
H	4.350899	0.430340	-0.094891
H	3.683927	-0.115224	1.445091
H	4.771705	-2.337890	1.137773
H	5.415847	-1.819853	-0.414179
H	7.146962	-1.593852	1.378895
H	6.730848	-0.002762	0.728979
H	6.080067	-0.517800	2.290796
H	1.626454	0.420335	1.541273
H	0.113339	0.759341	0.617338
H	-0.686315	-1.484498	0.946461
H	0.838181	-2.060054	1.642389
H	0.575629	-0.626550	3.588294
H	-0.771959	0.231715	2.857974
H	-1.573169	-1.455572	4.542042
H	-2.180768	-1.819088	2.922740
H	-0.831823	-2.753414	3.593786
H	1.060065	-0.665080	-2.436912
H	-0.297654	-0.373743	-1.345713
H	1.406621	-2.945609	-1.739661
H	0.299355	-2.802082	-0.379282

H	-1.594847	-2.396663	-1.910220
H	-0.522409	-2.437035	-3.298264
H	-1.683344	-4.640086	-3.010457
H	0.061125	-4.836755	-2.793530
H	-1.008786	-4.797023	-1.384221
C	-2.289352	1.608150	-0.741349
C	-2.752923	0.464050	0.178440
O	-0.927987	1.519810	-0.796388
H	-2.560659	-0.504963	-0.292117
H	-2.230141	0.525538	1.133380
Br	-4.657717	0.456146	0.649130
C	-2.941126	1.489296	-2.124071
H	-2.557346	2.289141	-2.763824
H	-2.655514	0.529469	-2.577360
H	-4.034283	1.552360	-2.088986
H	-2.652584	2.552419	-0.279136

Zero-point correction= 0.597040 (Hartree/Particle)
Thermal correction to Energy= 0.627272
Thermal correction to Enthalpy= 0.628216
Thermal correction to Gibbs Free Energy= 0.531811
Sum of electronic and zero-point Energies= -3450.054843
Sum of electronic and thermal Energies= -3450.024612
Sum of electronic and thermal Enthalpies= -3450.023668
Sum of electronic and thermal Free Energies= -3450.120073

SCF-energy at M06-2X/6-311+G(d,p)
$\mathbf{E_{scf}}$= -3453.2119641au

TS2s

Cartesian coordinates and thermal corrections at M06-2X/6-31+G(d,p) in gas phase

C	0.158376	-1.088300	0.555480
C	0.328973	-2.438592	-0.125564
C	-0.921083	-3.281570	0.146577
C	-0.792988	-4.688047	-0.432143
C	2.283189	-0.093957	1.410559
C	2.964762	-1.438687	1.619830
C	4.078017	-1.296596	2.661799
C	4.765649	-2.628348	2.952382
C	1.830133	-0.206634	-1.056869
C	2.745023	0.892825	-1.574731
C	3.356084	0.440724	-2.904535
C	4.212634	1.528723	-3.546117
C	0.512156	1.314194	0.426189

C	1.312277	2.550915	0.822435
C	0.395204	3.772148	0.694603
C	1.068746	5.052098	1.181538
N	1.243330	-0.025422	0.325360
H	0.057717	-1.179081	1.641586
H	-0.763565	-0.642702	0.185329
H	0.403169	-2.306502	-1.208901
H	1.221505	-2.976094	0.215024
H	-1.114181	-3.334551	1.226146
H	-1.777562	-2.771902	-0.311435
H	-1.705513	-5.266765	-0.268027
H	-0.611012	-4.646004	-1.510650
H	0.037657	-5.231176	0.030787
H	1.767455	0.202680	2.329859
H	3.029626	0.671611	1.185654
H	2.240609	-2.181526	1.970005
H	3.396149	-1.817066	0.685701
H	4.816930	-0.567819	2.305899
H	3.657493	-0.888097	3.589085
H	5.558719	-2.509630	3.694526
H	4.050593	-3.360830	3.339530
H	5.213679	-3.044284	2.044336
H	2.364126	-1.159189	-1.043413
H	0.966891	-0.302230	-1.721996
H	2.173163	1.811924	-1.745650
H	3.552925	1.128074	-0.870287
H	3.961553	-0.459172	-2.737494
H	2.547944	0.153547	-3.588256
H	4.645036	1.184767	-4.488949
H	3.615259	2.420950	-3.757778
H	5.035830	1.823230	-2.886665
H	-0.267824	1.157246	1.177721
H	0.032865	1.442099	-0.550587
H	1.645962	2.475811	1.863556
H	2.203106	2.701751	0.205973
H	0.091586	3.885417	-0.353720
H	-0.522587	3.593858	1.269020
H	0.402981	5.911966	1.074899
H	1.344223	4.971650	2.237856
H	1.979911	5.259320	0.610488
C	-3.575588	-0.446148	0.166911
C	-3.037107	0.746934	-0.459303
O	-4.038812	0.614207	1.018971
H	-3.664153	1.317969	-1.131048

H	-2.146636	1.193491	-0.047616
Br	-1.577988	-0.130852	-2.350032
C	-4.680272	-1.196374	-0.524957
H	-5.193969	-1.854139	0.180235
H	-5.406297	-0.498535	-0.949305
H	-4.249364	-1.794173	-1.333743
H	-2.840201	-1.073664	0.669255
C	-3.119431	0.916215	2.342439
O	-3.770639	1.568460	3.105527
O	-2.007308	0.423135	2.201437

Zero-point correction= 0.611476 (Hartree/Particle)
Thermal correction to Energy= 0.644700
Thermal correction to Enthalpy= 0.645645
Thermal correction to Gibbs Free Energy= 0.543729
Sum of electronic and zero-point Energies= -3638.560620
Sum of electronic and thermal Energies= -3638.527396
Sum of electronic and thermal Enthalpies= -3638.526452
Sum of electronic and thermal Free Energies= -3638.628368
Imaginary frequency: 537.8675 cm^{-1}
IR Intensity: 1196.4573

SCF-energy at M06-2X/6-311+G(d,p)
$\mathbf{E_{scf}}$= -3641.780417au

A2s

Cartesian coordinates and thermal corrections at M06-2X/6-31+G(d,p) in gas phase

C	0.743460	0.994663	-0.492279
C	0.401382	1.954298	0.639027
C	-0.984262	2.544074	0.356820
C	-1.507847	3.363258	1.533020
C	3.124758	0.786276	-1.202383
C	3.521210	2.200632	-0.805058
C	4.596049	2.734954	-1.755545
C	5.035726	4.149706	-1.386477
C	2.422337	0.015888	1.076804
C	3.608955	-0.884192	1.387980
C	3.963976	-0.751581	2.871986
C	5.107667	-1.679567	3.273492
C	1.667810	-1.205908	-0.971483
C	2.796001	-2.063496	-1.536990
C	2.261011	-3.476751	-1.789888
C	3.285399	-4.361344	-2.495238
N	2.033974	0.157835	-0.381871

H	0.823274	1.521181	-1.450087
H	-0.063882	0.265542	-0.569464
H	0.334703	1.399115	1.578070
H	1.136643	2.760528	0.755980
H	-0.957584	3.159273	-0.552325
H	-1.674284	1.715604	0.159589
H	-2.484238	3.800433	1.306597
H	-1.618393	2.728665	2.417752
H	-0.824265	4.182155	1.782727
H	2.763461	0.773869	-2.236745
H	3.994561	0.128740	-1.151252
H	2.652750	2.867644	-0.834201
H	3.910473	2.219137	0.219851
H	5.463156	2.062611	-1.741713
H	4.209761	2.724564	-2.782365
H	5.796770	4.520515	-2.077370
H	4.188836	4.842494	-1.416577
H	5.456026	4.178303	-0.376212
H	2.618066	1.027058	1.439245
H	1.521450	-0.366554	1.564144
H	3.353760	-1.929633	1.184167
H	4.494309	-0.633503	0.788429
H	4.237495	0.289096	3.088093
H	3.074548	-0.973561	3.473108
H	5.349804	-1.573268	4.333908
H	4.842276	-2.726135	3.093836
H	6.013415	-1.459236	2.698537
H	0.953020	-1.000325	-1.772821
H	1.096347	-1.703804	-0.181057
H	3.156710	-1.645913	-2.485323
H	3.660607	-2.126852	-0.870496
H	1.973151	-3.924380	-0.831332
H	1.342492	-3.418139	-2.386080
H	2.896791	-5.371484	-2.646552
H	3.547766	-3.952867	-3.476748
H	4.206902	-4.440608	-1.908655
C	-3.818263	-0.950931	0.826294
C	-3.996715	0.267225	-0.073937
O	-2.588415	-0.794527	1.525699
H	-4.001430	1.183214	0.520796
H	-3.217149	0.290423	-0.832291
Br	-5.687896	0.231620	-1.035090
C	-4.894870	-1.084106	1.888938
H	-4.652475	-1.923454	2.544510

H	-4.937907	-0.173844	2.496314
H	-5.873729	-1.252177	1.435831
H	-3.773806	-1.845868	0.196078
C	-1.407506	-1.052238	0.828606
O	-1.490550	-1.289322	-0.395379
O	-0.384699	-0.968059	1.539830

Zero-point correction= 0.614372 (Hartree/Particle)
Thermal correction to Energy= 0.647210
Thermal correction to Enthalpy= 0.648154
Thermal correction to Gibbs Free Energy= 0.545573
Sum of electronic and zero-point Energies= -3638.602451
Sum of electronic and thermal Energies= -3638.569613
Sum of electronic and thermal Enthalpies= -3638.568669
Sum of electronic and thermal Free Energies= -3638.671249

SCF-energy at M06-2X/6-311+G(d,p)
$\mathbf{E_{scf}}$= -3641.8315984au

TS3s

Cartesian coordinates and thermal corrections at M06-2X/6-31+G(d,p) in gas phase

C	-0.768270	-1.023230	-1.333531
C	-0.219914	-2.151391	-0.467410
C	0.893026	-2.880539	-1.230169
C	1.660989	-3.841420	-0.324723
C	-3.256072	-0.771669	-1.164281
C	-3.483058	-2.240485	-0.833509
C	-4.919682	-2.638981	-1.184535
C	-5.182986	-4.117768	-0.910780
C	-1.764750	-0.066465	0.724585
C	-2.564846	1.007668	1.442686
C	-2.184837	0.986590	2.926870
C	-2.896546	2.089571	3.705281
C	-1.773342	1.188810	-1.437849
C	-3.012953	2.074799	-1.538403
C	-2.558930	3.503357	-1.855923
C	-3.739492	4.448992	-2.057974
N	-1.925191	-0.183993	-0.779054
H	-1.115372	-1.395960	-2.303894
H	0.043341	-0.311444	-1.490974
H	0.213058	-1.734474	0.447107
H	-0.985288	-2.872270	-0.162892
H	0.459513	-3.420516	-2.082046
H	1.591153	-2.143978	-1.649743

H	2.423355	-4.384486	-0.887910
H	2.180930	-3.299629	0.472996
H	0.985965	-4.573730	0.131610
H	-3.346384	-0.623015	-2.245769
H	-4.018802	-0.156969	-0.679920
H	-2.790162	-2.871994	-1.400527
H	-3.310355	-2.439146	0.230307
H	-5.619317	-2.024485	-0.604780
H	-5.111507	-2.418944	-2.242254
H	-6.212388	-4.387986	-1.157711
H	-4.517123	-4.750661	-1.505582
H	-5.017353	-4.355254	0.144773
H	-1.981234	-1.054587	1.137957
H	-0.705799	0.140209	0.865144
H	-2.295533	1.992985	1.051791
H	-3.647902	0.859105	1.335709
H	-2.419195	0.005663	3.361371
H	-1.099957	1.129096	3.008425
H	-2.624726	2.062442	4.763329
H	-2.617810	3.071844	3.312253
H	-3.984824	1.987316	3.633888
H	-1.410777	0.982744	-2.449639
H	-0.973399	1.688776	-0.881059
H	-3.684538	1.720005	-2.329878
H	-3.591400	2.089900	-0.611437
H	-1.919909	3.857409	-1.038114
H	-1.931921	3.494400	-2.756671
H	-3.397753	5.464680	-2.271668
H	-4.369070	4.123689	-2.892963
H	-4.366193	4.489531	-1.161028
C	2.547401	0.492283	1.951096
C	2.701341	-0.029900	0.526723
O	1.816270	1.733081	1.929302
H	2.224632	-0.942811	0.211655
H	3.325287	0.493441	-0.181433
Br	4.578708	-1.476335	0.985517
C	1.820753	-0.494514	2.850809
H	1.683716	-0.062526	3.844646
H	0.829535	-0.723978	2.437868
H	2.400037	-1.417185	2.927693
H	3.528509	0.737064	2.355286
C	0.960513	1.821982	0.878195
O	1.163812	0.917690	-0.020235
O	0.080355	2.674007	0.870055

Zero-point correction=	0.614148 (Hartree/Particle)
Thermal correction to Energy=	0.646228
Thermal correction to Enthalpy=	0.647173
Thermal correction to Gibbs Free Energy=	0.547370
Sum of electronic and zero-point Energies=	-3638.575832
Sum of electronic and thermal Energies=	-3638.543752
Sum of electronic and thermal Enthalpies=	-3638.542808
Sum of electronic and thermal Free Energies=	-3638.642611

Imaginary frequency: 503.2989 cm^{-1}

IR Intensity: 1343.4080

SCF-energy at M06-2X/6-311+G(d,p)

$\mathbf{E_{scf}}$= -3641.80561294au
